# E3 ubiquitin ligase MAGI3 degrades c-Myc and acts as a predictor for chemotherapy response in colorectal cancer

**DOI:** 10.1186/s12943-022-01622-9

**Published:** 2022-07-22

**Authors:** Haibo Wang, Wenjing Yang, Qiong Qin, Xiaomei Yang, Ying Yang, Hua Liu, Wenxiu Lu, Siyu Gu, Xuedi Cao, Duiping Feng, Zhongtao Zhang, Junqi He

**Affiliations:** 1 Beijing Key Laboratory for Tumor Invasion and Metastasis, Department of Biochemistry and Molecular Biology, Capital Medical University, No.10 Xitoutiao, You An Men, Beijing, 100069 People’s Republic of China; 2 Department of Oncology, Beijing Hospital of Traditional Chinese Medicine, Capital Medical University, Beijing, People’s Republic of China; 3 Core Facilities Center, Capital Medical University, Beijing, People’s Republic of China; 4 Department of Interventional Radiology, First Hospital of Shanxi Medical University, Taiyuan, People’s Republic of China; 5 Department of General Surgery, Beijing Friendship Hospital, Capital Medical University & National Clinical Research Center for Digestive Diseases, No.95 Yong-anRoad, Xi-Cheng District, Beijing, 100050 People’s Republic of China

**Keywords:** MAGI3, c-Myc, Colorectal cancer, PDZ, Recurrence, Fluoropyrimidine-based systemic chemotherapy

## Abstract

**Background:**

Recurrence and chemoresistance constitute the leading cause of death in colorectal cancer (CRC). Thus, it is of great significance to clarify the underlying mechanisms and identify predictors for tailoring adjuvant chemotherapy to improve the outcome of CRC.

**Methods:**

By screening differentially expressed genes (DEGs), constructing random forest classification and ranking the importance of DEGs, we identified membrane associated guanylate kinase, WW and PDZ domain containing 3 (*MAGI3*) as an important gene in CRC recurrence. Immunohistochemical and western blot assays were employed to further detect MAGI3 expression in CRC tissues and cell lines. Cell counting kit-8, plate colony formation, flow cytometry, sub-cutaneous injection and azoxymethane plus dextran sulfate sodium induced mice CRC assays were employed to explore the effects of MAGI3 on proliferation, growth, cell cycle, apoptosis, xenograft formation and chemotherapy resistance of CRC. The underlying molecular mechanisms were further investigated through gene set enrichment analysis, quantitative real-time PCR, western blot, co-immunoprecipitation, ubiquitination, GST fusion protein pull-down and immunohistochemical staining assays.

**Results:**

Our results showed that dysregulated low level of MAGI3 was correlated with recurrence and poor prognosis of CRC. MAGI3 was identified as a novel substrate-binding subunit of SKP1-Cullin E3 ligase to recognize c-Myc, and process c-Myc ubiquitination and degradation. Expression of MAGI3 in CRC cells inhibited cell growth, promoted apoptosis and chemosensitivity to fluoropyrimidine-based chemotherapy by suppressing activation of c-Myc in vitro and in vivo. In clinic, the stage II/III CRC patients with MAGI3-high had a significantly good recurrence-free survival (~ 80%, 5-year), and were not necessary for further adjuvant chemotherapy. The patients with MAGI3-medium had a robustly good response rate or recurrence-free survival with fluoropyrimidine-based chemotherapy, and were recommended to undergo fluoropyrimidine-based adjuvant chemotherapy.

**Conclusions:**

MAGI3 is a novel E3 ubiquitin ligase by degradation of c-Myc to regulate CRC development and may act as a potential predictor of adjuvant chemotherapy for CRC patients.

**Graphical Abstract:**

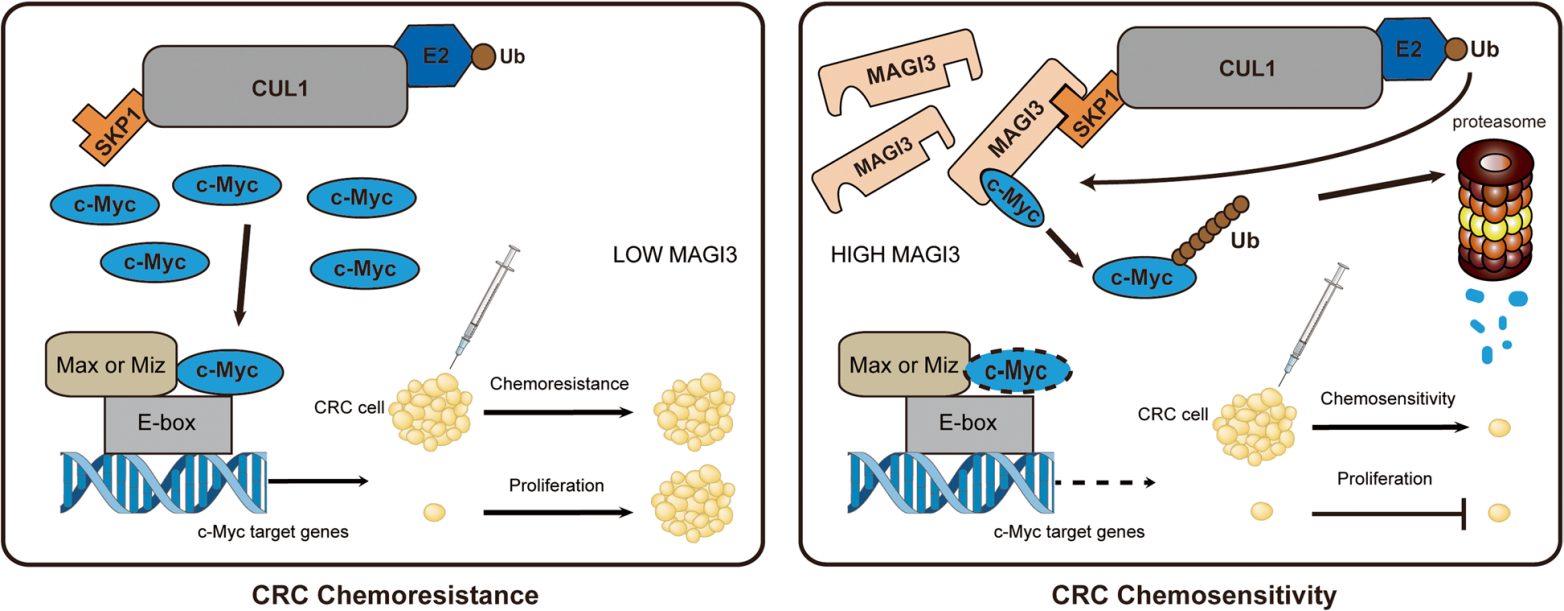

**Supplementary Information:**

The online version contains supplementary material available at 10.1186/s12943-022-01622-9.

## Background

Colorectal cancer (CRC) is the third most common cancer and the fourth most common cause of cancer-related death, with an estimated 1.8 million new cases and 880,000 attributed deaths in 2018 [1]. The prognosis of CRC is related to the stage at diagnosis, with a 5-year survival rate of approximately 90% for stage I, 70% for stage II, 58% for stage III, and less than 15% for stage IV [2]. To improve the long-term survival rate, it is a standard treatment for patients to receive adjuvant chemotherapy, and fluoropyrimidine [5-fluorouracil (5-FU) or capecitabine]-based systemic adjuvant chemotherapy has been widely used to all stage III CRC patients, and stage II patients with high-risk clinicopathologic features [3, 4]. However, a recent meta-analysis of 25 studies with good quality of reporting find the 5-year recurrence-free survival (RFS) for patients with stage II disease are 82.7% without adjuvant chemotherapy and 79.3% with adjuvant chemotherapy; for stage III disease, the percentages are 49.0% and 63.6% [5], which means that only a few percent of patients benefit from adjuvant chemotherapy, exposing majority of patients to unnecessary toxicity [6]. Thereby, a better understanding of the molecular mechanisms of CRC development to identify a powerful predictor of outcome may seem crucial to tailor adjuvant treatment.

The carcinogenesis and development of CRC is a multi-step process involving numerous genomic alterations. Chromosomal instability, microsatellite instability (MSI), and CpG island methylator phenotype defects are mechanisms involved in colorectal epithelial cell transformation, leading to CRC initiation and progression [7, 8]. Some gene mutations including *APC*, *TP53*, *SMAD4*, *KRAS*, and several altered molecular signaling pathways are also involved in CRC onset, such as DNA mismatch repair, Wnt/β-catenin, TGF-β/Smad, RAS-MAPK, PI3K/AKT, NF-κb and c-Myc signaling [7–11]. The *c-Myc* gene is one of the most common overexpressed genes in human tumors, and it is required for the maintenance and development of most cancers including CRC [12, 13].

All of the mentioned mutations and alterations during the initiation and progression of CRC could serve as biomarkers for CRC screening and prognostic evaluation. Microsatellite instability-high (MSI-H) in stage III responds better to immune checkpoint inhibitors. For patients in stage II, MSI-H shows a better prognosis but no beneficial effect of chemotherapy has been observed in this subgroup [14, 15]. *KRAS* mutational testing in metastatic CRC is part of a standard care to select patients to treatment targeting the EGFR as the presence of a *KRAS* mutation predicts for insensitivity to the anti-EGFR antibodies [16–18], and *BRAF* mutation also predicts a lack of benefit from anti-EGFR therapy [19]. There is no evidence so far that CRC patients with *KRAS* or *BRAF*-mutated tumors are especially likely to benefit from standard chemotherapy agents [20]. Currently pathological staging is the main prognostic classification used in clinical practice to stratify patients for adjuvant chemotherapy [21, 22]. However, about 10% ~ 20% of patients with stage II CRC and 30% ~ 40% of those with stage III CRC develop recurrence, so pathological staging prediction is far from accuracy to meet clinical demands. Therefore, it is of great significance to clarify the mechanisms of carcinogenesis and recurrence to offer opportunities to identify a potential predictor to tailor adjuvant chemotherapy.

In this study, we screened differentially expressed genes (DEGs) in CRC databases from The Cancer Genome Atlas (TCGA) and Gene Expression Omnibus (GSE40967) to gain a panoramic view of expression patterns from tumor vs. adjacent noncancerous tissues and recurrence vs. non-recurrence tumors. Membrane associated guanylate kinase, WW and PDZ domain containing 3 (*MAGI3*) was identified as a novel independent prognostic marker of CRC patients, with dysregulated low level of MAGI3 correlated with recurrence and poor prognosis. We further provided the convincing evidence that MAGI3 is a novel substrate-binding subunit of SKP1-Cullin E3 ligase to recognize c-Myc, and process c-Myc ubiquitination and degradation. Dysregulated low level of MAGI3 in CRC promoted cell growth and reduced chemosensitivity to fluoropyrimidine-based chemotherapy by promoting activation of c-Myc in vitro and in vivo. In clinical studies, among the stage II/III CRC patients who do not receive adjuvant chemotherapy, patients with MAGI3-high had a significantly good RFS (~ 80% with 5-year RFS), and were not necessary for further adjuvant chemotherapy. Moreover, for the patients receiving adjuvant chemotherapy, those with MAGI3-medium had a significantly good response rate or RFS with fluoropyrimidine-based chemotherapy, and were recommended to undergo fluoropyrimidine-based adjuvant chemotherapy. The patients with MAGI3-low needed comprehensive treatment including targeted therapy or chemotherapy other than fluoropyrimidine. Taken together, these results suggest that MAGI3 is a novel E3 ubiquitin ligase by degradation of c-Myc to regulate CRC development and may act as a potential predictor of adjuvant chemotherapy for CRC patients.

## Methods

### Data collection and CRC samples

The genomic data and clinical data of CRC from TCGA (https://www.cbioportal.org/), GSE40967 and GSE72970 (https://www.ncbi.nlm.nih.gov/geo/query/acc.cgi?acc=GSE40967; GSE72970) were retrieved. The RNA sequencing data from TCGA, GSE40967 and GSE72970 included 375, 566 and 124 samples respectively. Human CRC samples, obtained from First Hospital of Shanxi Medical University (Shanxi Medical University, China) during surgical resection (*n* = 112), were collected from 2011 to 2014. None of these patients received preoperative chemotherapy or radiotherapy. The study of human CRC samples was approved by the Ethics Committee of First Hospital of Shanxi Medical University, and all of patients provided informed consent.

### Ranking of DEGs importance

Random forest is a popular classification and regression method that has been proven powerful for various prediction problems in biological studies [23–25]. Mean Decrease Accuracy (MDA), which is involved in the random forest algorithm, is used to rank the important indexes of DEGs. The MDA provides ways to quantify which index contributes most to classification accuracy. A higher MDA indicates that the degree of impurity arising from the category could be reduced farthest by one variable, thus it suggests an important associated index. Statistical analysis is performed using random Forest package of R software (http://www.r-project.org). The specific random forest model parameters were as follows: max features: auto, n estimators: 5000, min sample leaf: 1, and number of variables tried at each split: 4.

### Co-immunoprecipitation (CoIP)

The method used for immunoprecipitation has been described previously [26]. Briefly, cells were harvested and lysed in lysis buffer (10 mM Hepes, 50 mM NaCl, 5 mM EDTA, 1 mM benzamidine, 0.5% Triton X-100). After the lysate was solubilized and clarified, it was incubated with 3 µl antibody and 50 µl protein A/G-agarose for 3 h with end-over-end rotation at 4 °C. After washing three times in 1 ml ice-cold PBS buffer, the immunoprecipitated proteins were eluted from the beads with SDS-PAGE sample buffer and visualized by western blot analysis.

### In vivo xenograft formation and azoxymethane (AOM) / dextran sulfate sodium (DSS) colorectal tumor formation assays

All animal experiments were performed following the National Institutes of Health Guide for the Care and Use of Laboratory Animals and were approved by the Animal Use and Care Committee of Capital Medical University.

RKO cells (5 × 10^6^ cells in 0.1 ml PBS) stable transfected with MAGI3 (RKO-MAGI3) or pcDNA3.0 control (RKO-pcDNA3.0) were subcutaneously implanted into the flank in each of BALB/c nude mice (4–5 weeks old, weight 16-18 g, male, and 4 mice per group). The mice were monitored every two days for the growth of tumors, and they were killed after 15 days. The tumor xenografts were dissected and weighed after the sacrifice of the mice. Tumor volumes were estimated according to the equation: volume (mm^3^) = (length × width^2^)/2.

AOM/DSS colorectal tumor formation was induced in C57BL/6 mice (5–6 weeks old, weight 18-20 g, male, *n* = 4). Mice were injected intraperitoneally with 10 mg/kg AOM (A5486; Sigma-Aldrich, St. Louis, MO). Seven days after AOM injection, these mice were provided with 2.5% DSS (MB5535; Dalian Meilun Biotechnology Co., Ltd, China) in their drinking water for 7 consecutive days, followed by another 14 days of recovery. This round was repeated triple until day 70, when all mice were sacrificed and their whole colons were collected.

*MAGI3* knockout mice (*MAGI3*^−/−^) were generated by Shanghai Model Organisms Center, Inc (Shanghai, China). Genotyping of wild-type and knockout mice was performed with the following primers: forward primer 5’-ATCGCTTGCTTTTCTGAGTGTA-3’ and reverse primer 5’-GGCTTGGGTGTGCCATAGAA-3’ for wild-type mice; forward primer 5’-GAACACTTTCCCATGGTGCC-3’ and reverse primer 5’-CCAAACCAGAGGCCAGGAAT-3’ for *MAGI3*^−/−^ mice. All mice were on the C57BL/6 background and were maintained under specific-pathogen-free conditions. *MAGI3*^−/−^ mice and matched wild-type mice received modified AOM/DSS protocol (5–6 weeks old, weight 18-20 g, male, *n* = 4). Mice were injected intraperitoneally with 10 mg/kg AOM. Seven days after AOM injection, these mice were provided with 2.5% DSS in their drinking water for 7 consecutive days. All mice were sacrificed and their whole colons were collected at day 66. Disease activity index (DAI) score is the sum of stool consistency change (0, none; 2, loose stool; and 4, diarrhea), bleeding (0, none; 1, trace; 2, mild hemoccult; 3, obvious hemoccult; and 4, gross bleeding) and weight loss (0, none; 1, 0–5%; 2, 5–10%; 3, 10–20%; and 4, > 20%) and then divided by three. Mice were scored for the DAI at the same time of each day, and DAI score was recorded every three days.

### Glutathione S-transferase (GST) fusion protein pull-down assay

GST pull-down assay was described previously [27]. In brief, GST-c-Myc-wt, GST-c-Myc-∆ct and GST-SKP1 fusion proteins were induced and purified from bacteria (BL21) according to the manufacturer’s protocol, and then re-suspended in PBS containing 0.5% Nonidet P-40 and protease inhibitors. Equal amounts of GST fusion proteins (conjugated on beads) were incubated with 1 ml cell lysates with end-over-end rotation at 4˚C for 4 h. The beads were washed 4 times with washing buffer I (10 mM HEPES, 50 mM NaCl, 5 mM EDTA, 1 mM benzamidine, 0.1% Tween 20, and 3% bovine serum albumin), then washed 1 time with washing buffer II (buffer I without 3% bovine serum albumin). The proteins were eluted from the beads with 2 × SDS-PAGE sample buffer, resolved via SDS-PAGE, and detected by western blotting.

### Statistics analysis

All experiments were repeated at least 3 times. Data were statistical analyzed by SPSS 16.0 (IBM, Armonk, NY, USA) and GraphPad Prism software (GraphPad, CA, USA). Continuous variables were analyzed by analysis of variance or t-test, and nonparametric test was used if the data did not assume Gaussian distribution. A chi-square test or fisher exact test was used to compare the distribution differences between the two groups. Pearson correlation was employed to assess the correlation of variables, and spearman correlation was used if the data did not assume Gaussian distribution. The Kaplan–Meier (KM) survival curves and log-rank test were used to determine the differences in survival rates between two groups. Cox proportional hazards model was employed to perform univariate and multivariate analysis. Data were presented as mean ± SEM. *P* < 0.05 was considered statistically significant, and one or two-tailed *P*-value was used in analyses.

### Additional methods

More details regarding the methods can be found in supplementary materials (Supplementary materials and methods).

## Results

### Low level of MAGI3 is correlated with recurrence in CRC

**Fig. 1:**
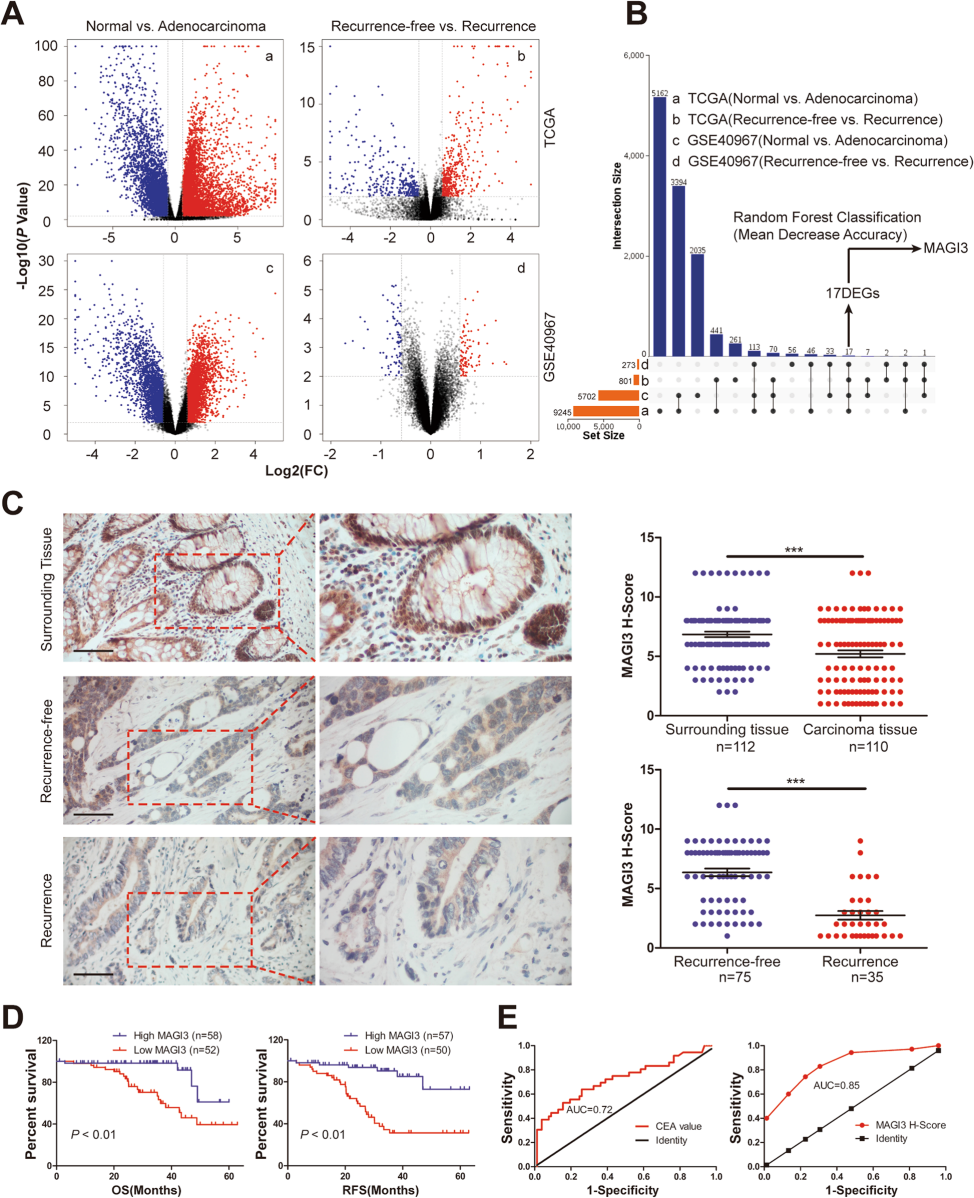
MAGI3 expression is downregulated in CRC of primary and recurrence tissues. **a**, DEGs correlated with CRC carcinogenesis and recurrence. DEGs of tumor vs. adjacent noncancerous tissues, and recurrence vs. non-recurrence tumors in CRC patients from TCGA and GSE40967 were displayed by the volcano plot. The horizontal gray line represented *P* = 0.01. The vertical gray lines showed 1.5-fold changes in gene expression. Red dots represented upregulated genes and blue dots represented downregulated genes. **b**, *MAGI3* was one of 17 DEGs identified in 2 independent datasets. The UpSetVenny diagram showed the 17 overlapped DEGs, and the importance of *MAGI3* ranked the top one in these DEGs by random forest classifier models analysis. **c**, The protein level of MAGI3 was also robustly reduced in CRC and recurrence specimens. Representative images of MAGI3 IHC staining in adjacent tissues (top), non-recurrence tumors (middle) and recurrence tumors (bottom) of CRC. Scale bars: 50 μm. Right panels are × 2 magnification of the dashed areas on the left. Dot plot showing the corresponding quantification of MAGI3 H-score. Data were presented as the mean ± SEM, and statistical significance was calculated by Mann–Whitney test. ***P < 0.001. **d**, patients with low MAGI3 protein had a worse OS and RFS. Kaplan–Meier curves showing comparison of the OS (left) and RFS (right) between MAGI3-low and -high expression groups of CRC patients (*P* < 0.01, calculated by log-rank test). **e**, MAGI3 was a potential predictor of postoperative recurrence in CRC patients. The receiver operating characteristic curve with serum carcinoembryonic antigen value (left) and MAGI3 in CRC specimens H-score (right)

To identify genes associated with CRC recurrence, a total of 17 DEGs were identified from two independent databases of TCGA and GSE40967 between tumor vs. adjacent noncancerous tissues, and recurrence vs. non-recurrence tumors (Fig. 1a and b), with *MAGI3* ranked in top one importance (Additional file 3: Table S1). The mRNA levels of *MAGI3* over 941 CRC cases were further found significantly downregulated in CRC or recurrence tissues (Additional file 4: Fig. S1A and B). Consistently, the protein level of MAGI3 was also robustly reduced in another cohort of 112 CRC specimens from a Chinese cohort, including 35 postoperative recurrence cases (Fig. 1c).

KM survival analysis revealed that patients with lower MAGI3 protein level had a worse overall survival (OS) and RFS than those with higher MAGI3 protein level (Fig. 1d). Cox univariate and multivariate analysis indicated that MAGI3 protein level was an independent factor for prognosis prediction of CRC patients (Additional file 3: Table S2). These results were also verified with *MAGI3* mRNA level (Additional file 3: Table S3; Additional file 4: Fig. S1C-G). To assess the efficacy of MAGI3 in predicting recurrence in CRC patients, we plotted the receiver operating characteristic (ROC) curve with MAGI3 protein level compared with that of serum carcinoembryonic antigen (CEA) which is the most common indicator used to predict CRC recurrence [28, 29]. The area under curve (AUC) of CEA was 0.72 ± 0.06 with the sensitivity 72% and specificity 61%, while the AUC of MAGI3 protein was 0.85 ± 0.04 with the sensitivity of 74% and specificity of 77% (Fig. 1e). Altogether, these findings demonstrate that low level of MAGI3 is significantly correlated with the poor prognosis of CRC patients, indicating that MAGI3 is a potential novel independent prognostic marker for CRC patients, and it may play important roles in CRC initiation and development.

### MAGI3 regulates proliferation and apoptosis of CRC cells

**Fig. 2:**
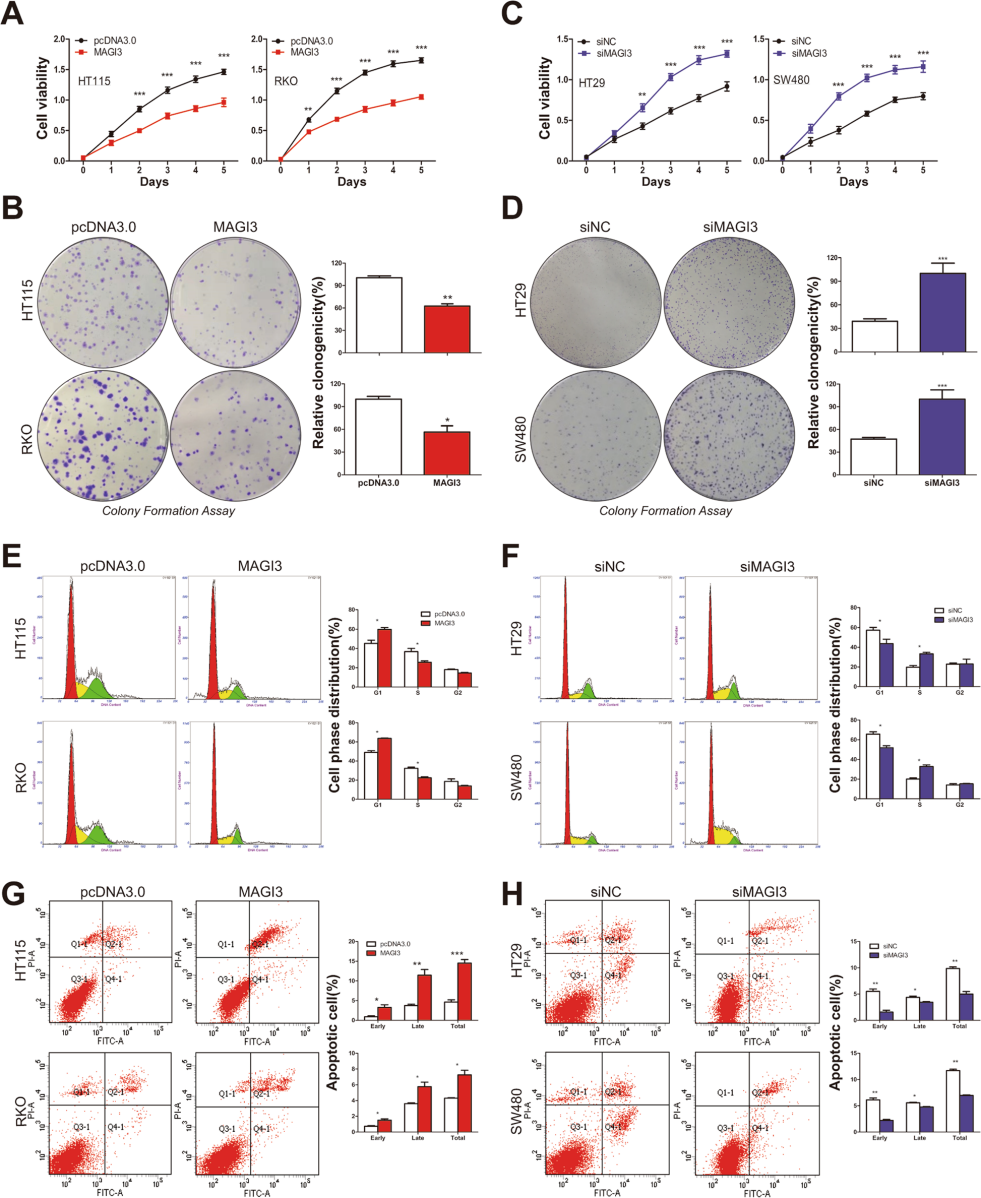
MAGI3 inhibits cell growth and induces cell cycle arrest and apoptosis in CRC cells. **a** and **b**, Overexpression of MAGI3 significantly inhibited CRC cell proliferation. Cell proliferation of HT115 or RKO cells, transfected with MAGI3 or pcDNA3.0, were assessed by CCK-8 (**a**) or colony formation assays (**b**). **c** and **d**, Knockdown of MAGI3 expression significantly enhanced CRC cell proliferation. Cell proliferation of HT29 or SW480 cells, transfected with siMAGI3 or siNC, were assessed by CCK-8 (**c**) or colony formation assays (**d**). **e** and **f**, MAGI3 induced cell cycle arrest. Flow cytometry assay showed that overexpression of MAGI3 induced cell cycle arrest at G1–S transition (**e**) and knockdown of MAGI3 promoted cell cycle progression (**f**). **g** and **h**, MAGI3 induced cell apoptosis. Flow cytometry assay showed that overexpression of MAGI3 induced CRC cell apoptosis (**g**) and knockdown of MAGI3 reduced CRC cell apoptosis (**h**). Q2-1 shows the late apoptotic cells and Q4-1 shows the early apoptotic cells. Data were presented as the mean ± SEM, 2-way repeated-measures ANOVA with Bonferroni post-test (**a** and **c**), 2-tailed, unpaired t test (**b**, **d**, **e**–**h**) were used to determine statistical significance. *P < 0.05, **P < 0.01, and ***P < 0.001. These experiments (**a**-**h**) were repeated three times independently

Tumor cell proliferation is a major representative indicator of malignant phenotype, so the effects of MAGI3 expression on CRC cell proliferation was investigated. First, HT115 and RKO CRC cell lines with low levels of MAGI3 (Additional file 4: Fig. S2A) were stably transfected with MAGI3 (Additional file 4: Fig. S2B), and the data showed that overexpression of MAGI3 significantly inhibited CRC cell proliferation (Fig. 2a) and colony formation (Fig. 2b). We further observed that knockdown of MAGI3 expression in HT29 or SW480 CRC cells with relatively high levels of MAGI3 (Additional file 4: Fig. S2A, C) significantly enhanced cell proliferation (Fig. 2c) and clonogenicity (Fig. 2d). Meanwhile, overexpression of MAGI3 significantly promoted CRC cell cycle arrest (Fig. 2e) with the changed expression of proliferation and cell cycle related proteins, such as PCNA, p21, cyclinD1, and CDK4 (Additional file 4: Fig. S2D). Conversely, knockdown of MAGI3 in HT29 or SW480 cells markedly decreased CRC cell cycle arrest (Fig. 2f). Furthermore, overexpression of MAGI3 increased CRC cell apoptosis (Fig. 2g) with activation of caspases and PARP (Additional file 4: Fig. S2E), and knockdown of MAGI3 reduced cell apoptosis in CRC cells (Fig. 2h). Taken together, our results suggest that MAGI3 acts as a tumor suppressor by regulating proliferation and apoptosis in CRC cells.

### MAGI3 modulates c-Myc degradation through the ubiquitin proteasome pathway and regulates c-Myc transcriptional target genes expression

**Fig. 3:**
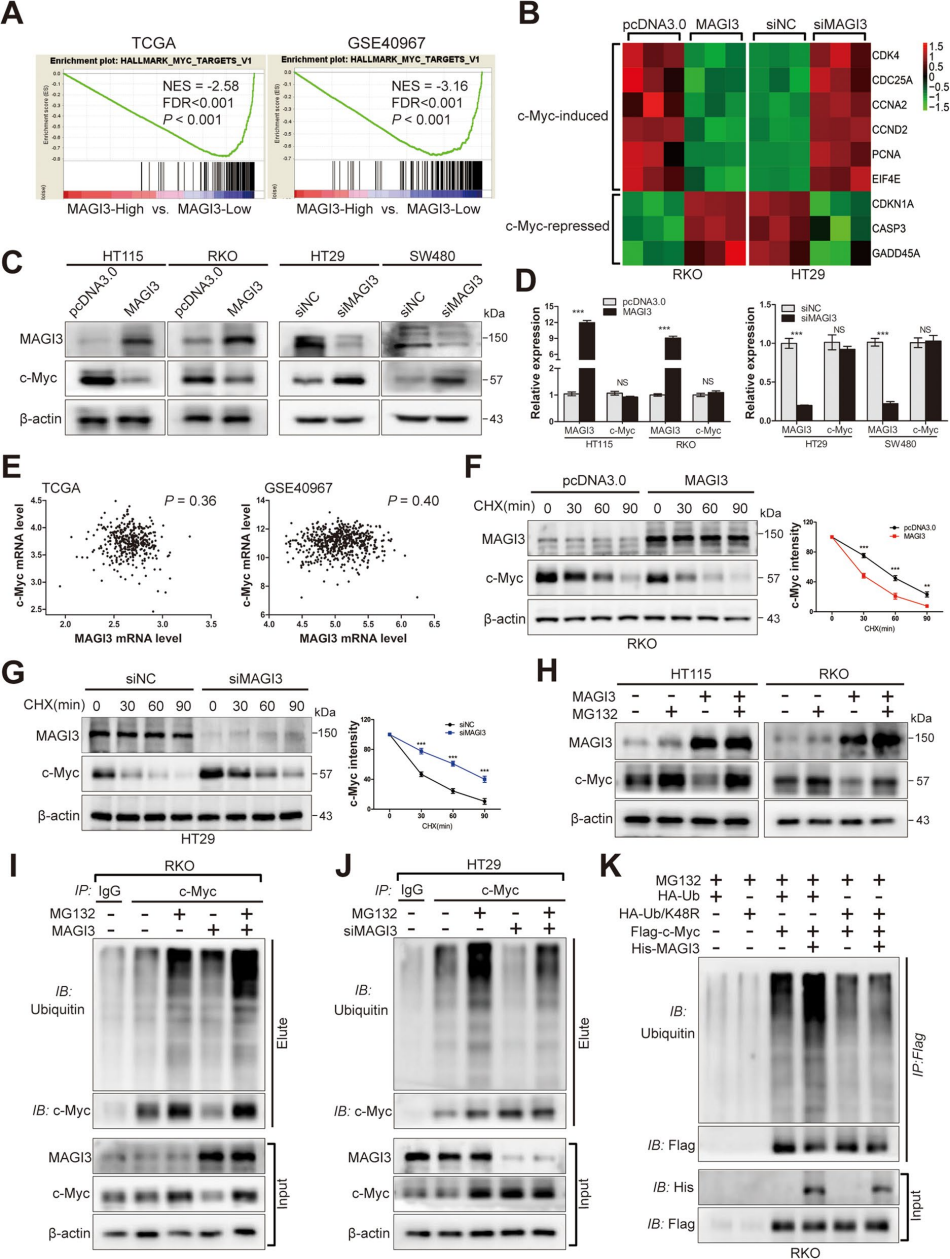
MAGI3 regulates c-Myc activation through degradation of c-Myc by the ubiquitin pathway. **a**, Enrichment plots of GSEA showed that the gene signatures of c-Myc targets were significantly enriched in low *MAGI3* expression CRC specimens. **b**, MAGI3 inhibited c-Myc transcriptional target genes expression. Heatmap of c-Myc target genes expression modulated by MAGI3 in RKO or HT29 cells (three independent experiments). **c**, Overexpression of MAGI3 decreased, whereas knockdown of MAGI3 increased the protein levels of c-Myc. c-Myc protein levels were detected by western blot in HT115 or RKO cells over-expressed MAGI3 (left), and in HT29 or SW480 cells silenced MAGI3 (right). **d**, MAGI3 had no detectable effect on the *c-Myc* expression at mRNA level. qPCR analysis of the *c-Myc* mRNA expression in RKO or HT29 cells with the indicated MAGI3 modulation, three independent experiments, data were presented as the mean ± SEM, a 2-tailed, unpaired t test was used to determine statistical significance. ***P < 0.001, NS, no significance. **e**, *MAGI3* and *c-Myc* had no correlation (by Pearson’s) at transcriptional level in CRC specimens from TCGA (left, *n* = 375) and GSE40967 (right, *n* = 566). **f** and **g**, Overexpression of MAGI3 decreased, whereas knockdown of MAGI3 prolonged the half-life of c-Myc. The effect of MAGI3 on the half-life of c-Myc were detected by western blot in CRC cells treated with CHX. The protein half-life curves were obtained by quantifying three independent experiments (right). **h**, MAGI3 regulated c-Myc turn over through proteasome pathway. The levels of c-Myc protein were detected by western blot in CRC cells transfected with MAGI3 and treated with MG132 (10 μM) for 6 h before harvesting. **i** and **j**, Overexpression of MAGI3 promoted, whereas knockdown of MAGI3 inhibited c-Myc ubiquitin. Ubiquitination assays of c-Myc in lysates from RKO or HT29 cells with the indicated MAGI3 manipulation. **k**, MAGI3 regulated c-Myc ubiquitin in Lys48-linked poly-ubiquitination. Ubiquitination assays of c-Myc in RKO cells transfected with HA-Ub or HA-Ub/K48R

To gain insight into the molecular mechanism of MAGI3 expression control cell functions, Gene Set Enrichment Analysis (GSEA) was performed. The results showed that the gene signatures of c-Myc targets were enriched in patients with *MAGI3* lower levels in TCGA CRC and GSE40967 datasets (Fig. 3a). The results were further verified in our cell studies that overexpression of MAGI3 in RKO cell downregulated the expression of a panel of genes classically promoted by c-Myc, such as *CDK4*, *CDC25A*, *CCNA2*, *CCND2*, *PCNA* and *EIF4E*, and upregulated the expression of a panel of genes classically inhibited by c-Myc, as *CDKN1A*, *CASP3* and *GADD45A*. Furthermore, the converse results were observed when MAGI3 was knockdown in HT29 cell (Fig. 3b). Taken together, these results indicate that MAGI3 inhibited c-Myc activation.

Our further experiments showed that MAGI3 could regulate c-Myc expression. Overexpression of MAGI3 in CRC cells markedly decreased c-Myc protein levels, whereas knockdown of MAGI3 in CRC cells significantly increased c-Myc protein levels (Fig. 3c). To explore the underlying mechanism, we first tested MAGI3 on *c-Myc* expression at transcriptional level. As the results shown, neither overexpression nor knockdown of MAGI3 had detectable effect on *c-Myc* mRNA levels (Fig. 3d), and there were no correlations between *MAGI3* and *c-Myc* mRNA in clinical specimens (Fig. 3e). We then examined the expression of MAGI3 on c-Myc protein stability, and found that the half-life of c-Myc protein was markedly decreased with MAGI3 overexpression (Fig. 3f and Additional file 4: Fig. S3A) and significantly increased by knockdown of MAGI3 in CRC cells (Fig. 3g and Additional file 4: Fig. S3B). c-Myc protein is reported to be mainly regulated by the ubiquitin proteasome pathway [30], so we investigated whether MAGI3 modulated c-Myc stability through proteasome pathway. We found that c-Myc protein levels were robustly increased in CRC cells overexpressed MAGI3 after treated with MG132, a proteasome inhibitor (Fig. 3h; lane 3, 4). Meanwhile, blocked proteasome activation with MG132, c-Myc protein levels were less affected by MAGI3 overexpression (Fig. 3h; lane 2, 4). These results indicate that MAGI3 regulates c-Myc expression through proteasome pathway. To confirm the results, the ubiquitination of c-Myc was detected in the presence or absence MAGI3. The enhanced c-Myc ubiquitination and reduced c-Myc protein level were observed in RKO cell overexpressed MAGI3 (Fig. 3i; lane 2, 4). Conversely, knockdown of MAGI3 reduced c-Myc ubiquitination and increased c-Myc protein level in HT29 cell (Fig. 3j; lane 2, 4). As expected, when cells were treated with MG132, a distinct c-Myc ubiquitination could be detected coordinately with MAGI3 manipulated, but no significant c-Myc protein levels were changed (Fig. 3i and j; lane 3, 5). Furthermore, we found that MAGI3 modulated c-Myc ubiquitin in Lys48-linked polyubiquitylation (Fig. 3k). Taken together, these data demonstrate that MAGI3 modulates c-Myc protein stability by promoting c-Myc ubiquitin–proteasome degradation, thereby regulates c-Myc signaling.

### MAGI3 is identified as a novel substrate-binding subunit of SKP1-Cullin E3 ligase to recognize c-Myc protein

**Fig. 4:**
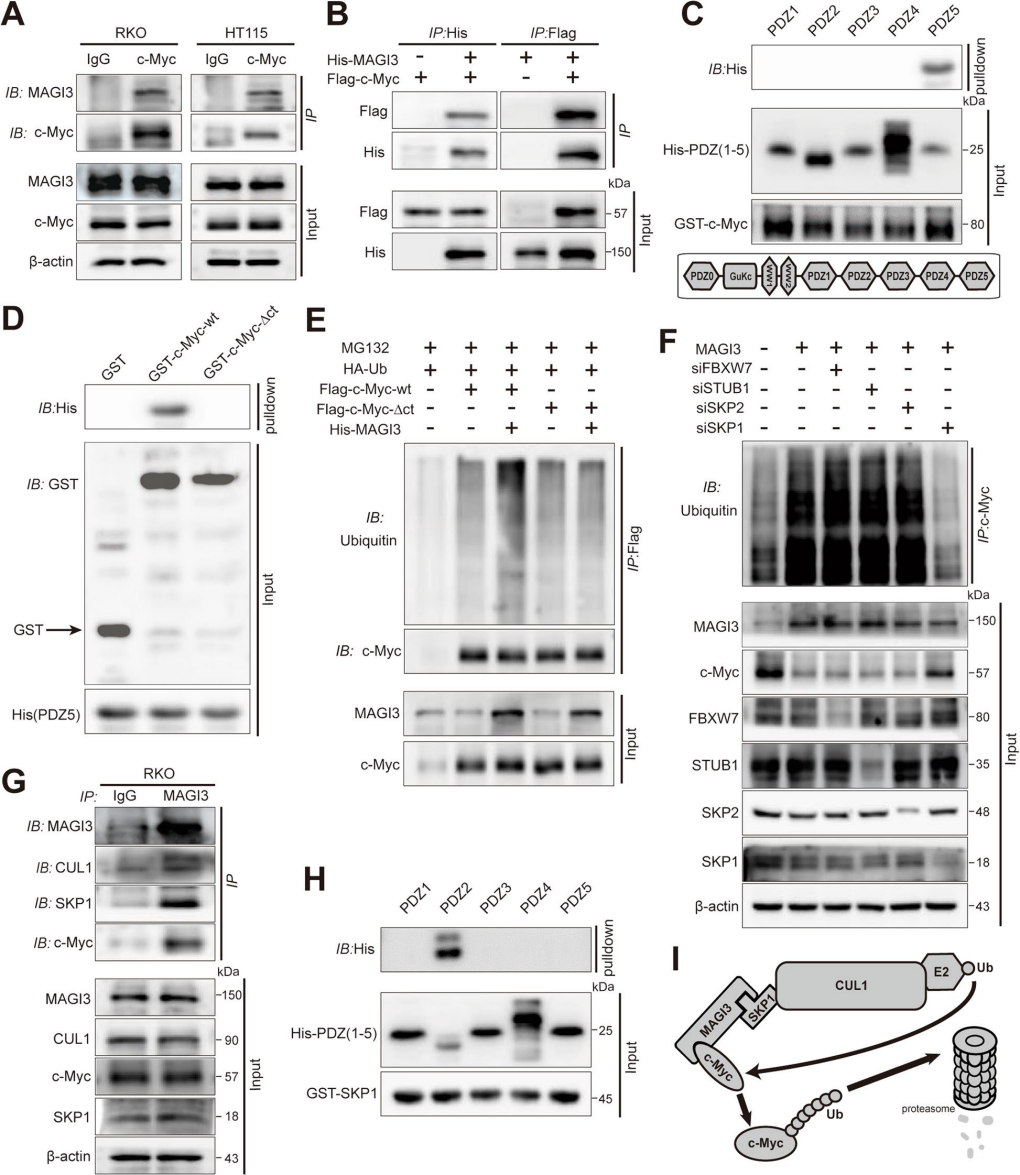
MAGI3 is a substrate-binding subunit of SKP1-Cullin E3 ligase to recognize c-Myc. **a** and **b**, MAGI3 was interacted with c-Myc. **a**, The cell lysates of CRC cells were immunopreciptated with anti-c-Myc antibodies, and MAGI3 was determined in the Co-IP by western blot. **b**, The reciprocal Co-IP was performed to detected the association of MAGI3 with c-Myc in HEK293 cells. **c**, The fifth PDZ domain of MAGI3 directly bound with the c-Myc. The individual His-PDZ domains of MAGI3 were pulled down with GST-c-Myc fusion proteins. The schematic representation of five PDZ domains of MAGI3 (bottom). **d**, The PBM in c-Myc was essential for the interaction of c-Myc with MAGI3. His-PDZ5 domain of MAGI3 was pulled with GST-c-Myc wild-type (GST-c-Myc-wt) or its deletion of PBM (GST-c-Myc-Δct). **e**, The interaction of MAGI3 with c-Myc was essential for MAGI3 regulation of c-Myc ubiquitination. Ubiquitination of c-Myc-wt or c-Myc-∆ct in RKO cells with the indicated MAGI3 manipulation were detected. Cells were treated with MG132 (10 μM) before harvesting. **f**, SKP1 is required for c-Myc ubiquitination and degradation caused by MAGI3 overexpression. Ubiquitination of c-Myc in RKO cell was detected after SKP1 and F-box proteins were silenced with the indicated siRNAs. **g**, MAGI3 interacted with c-Myc, SKP1 and CUL1 in RKO cell. The cell lysates of RKO-MAGI3 cell were immunopreciptated with anti-MAGI3 antibodies, and c-Myc, SKP1 and CUL1 were determined in CoIP complex by western blot. **h**, The second PDZ domain of MAGI3 directly bound with SKP1. The individual His-PDZ domains of MAGI3 were pulled with GST-SKP1 fusion proteins. **i**, Schematic representation showing the proposed mechanism of MAGI3 in the regulation of c-Myc ubiquitination and degradation. As a mediator, MAGI3 links c-Myc with SKP1, promotes the ubiquitination of c-Myc and its degradation through proteasome pathway

MAGI3 is a scaffold protein containing PDZ domain, and c-Myc constitutes a potential PDZ domain binding motif (PBM) at its C-terminus (N-S-C-A), so there is possible an interaction between MAGI3 and c-Myc. Our CoIP results confirmed the association between endogenous MAGI3 and c-Myc in CRC cells (Fig. 4a), and the association was further validated by reciprocal CoIP of the exogenous tagged protein in HEK293 cells (Fig. 4b). The GST-pulldown assays further clarified the structural determinants of the MAGI3/c-Myc interaction that the fifth PDZ domain of MAGI3 directly bound with the c-Myc (Fig. 4c), with the PBM of c-Myc is required for the interaction (Fig. 4d). Abolished this interaction, c-Myc ubiquitination was no more upregulated by MAGI3 (Fig. 4e). All together, these results indicate that the MAGI3/c-Myc interaction is essential for MAGI3 upregulation of c-Myc ubiquitination and degradation.

The SKP1-Cullin-F-box protein (SCF) ubiquitin-protein ligases are the most abundant E3 ubiquitin ligases contribute to c-Myc degradation. F-box proteins, such as FBXW7, SKP2 and STUB1, are well established substrate-recognition components of SCF E3 ligase to specifically target c-Myc [31–33]. However, knockdown of FBXW7, SKP2 or STUB1 had little effect on c-Myc ubiquitination caused by MAGI3, whereas knockdown of SKP1, an adaptor in SCF complex, dramatically eliminated c-Myc ubiquitination and degradation caused by MAGI3 overexpression (Fig. 4f), indicating that MAGI3 is a novel substrate-binding subunit assembling c-Myc with SKP1 to form an E3 ligase. It was further verified by robust signals of c-Myc, SKP1 and CUL1 detected in the MAGI3 CoIP complex (Fig. 4g), and MAGI3 directly interacted with SKP1 via its second PDZ domain (Fig. 4h). Thereby, as the schematic diagram shown (Fig. 4i), these results demonstrate the existence of a macromolecular complex c-Myc-MAGI3-SKP1-CUL1, and PDZ domain of MAGI3 alternates with F-box domain, mediates the SKP1-Cullin E3 ligase targeting c-Myc, and processes c-Myc proteasome degradation.

### MAGI3 inhibits CRC development through downregulation of c-Myc protein

**Fig. 5:**
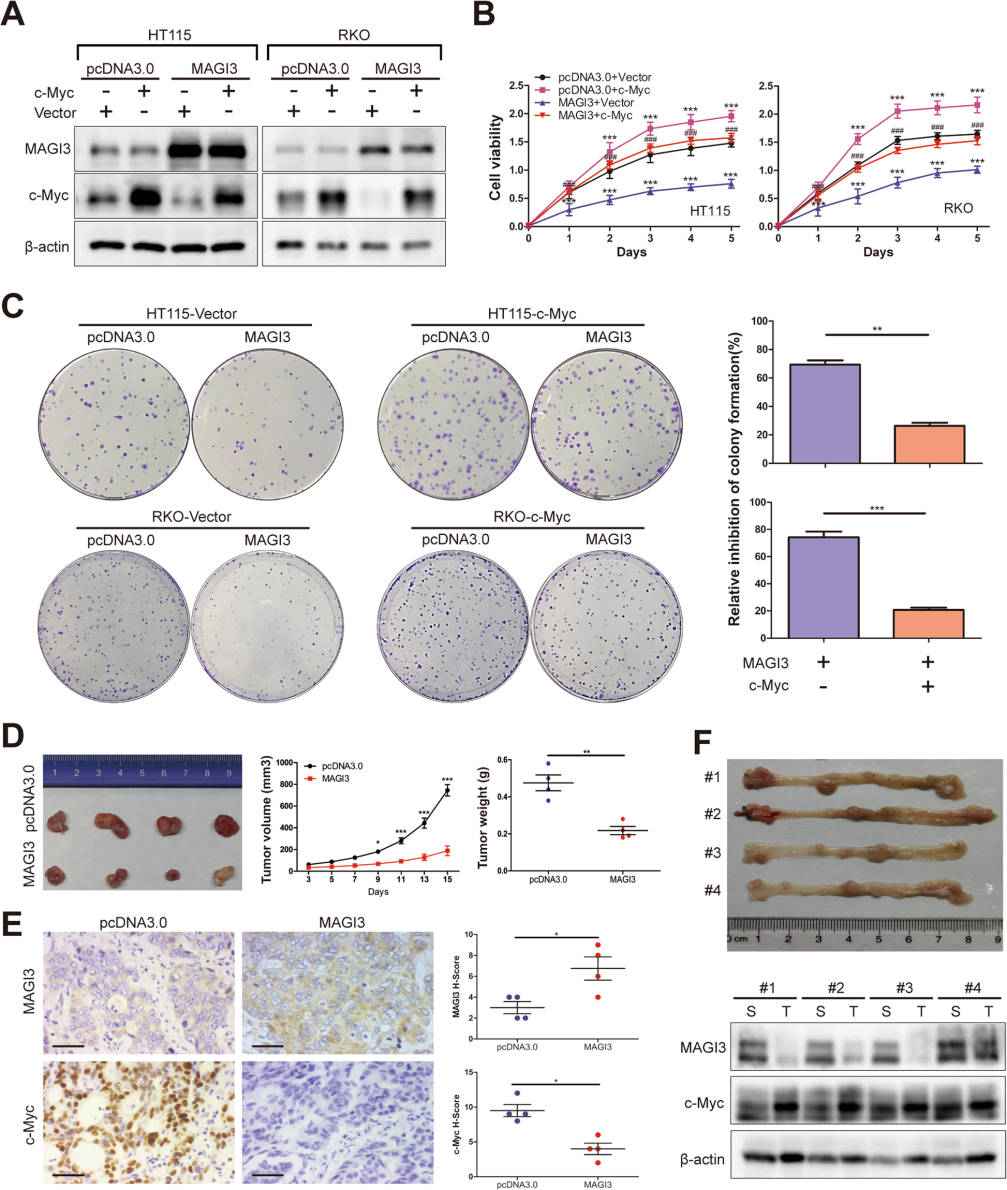
Suppression of CRC progression in vitro and in vivo by MAGI3 is dependent on c-Myc. **a**, To overexpression c-Myc in CRC-MAGI3 cells by exogenous transfection of c-Myc. c-Myc was overexpressed in HT115-MAGI3 or RKO-MAGI3 cells by transfected with c-Myc constructs. **b**, MAGI3 had less effect on the CRC cells proliferation when c-Myc protein levels were overexpressed. Cell viability of HT115 and RKO cells by CCK-8 assay. ***P < 0.001 vs. pcDNA3.0 + Vector; ^###^*P* < 0.001 vs. MAGI3 + Vector. **c**, Overexpressed c-Myc effectively restricted MAGI3-inhibition of colony formation of CRC cells. **d**, MAGI3 overexpression inhibited the growth of RKO xenografts in nude mice. Representative images of tumors from the implanted mice (left). The volume and weight of tumors were significantly decreased in MAGI3 overexpressed RKO xenograft tumors (middle and right). **e**, MAGI3 overexpression correlated with the reduced the expression of c-Myc in RKO xenografts. Representative IHC staining of MAGI3 and c-Myc in xenografted tumor. Scale bars: 50 μm. Dot plot (right) showed the corresponding quantification of MAGI3 or c-Myc H-score. **f**, Protein levels of MAGI3 were reduced coordinately with c-Myc increase in AOM/DSS induced CRC. Images of AOM/DSS induced CRC (top). The protein levels of MAGI3 and c-Myc in colorectal tumor (T) and matched surrounding tissues (S) were detected by western blot (bottom). Data were presented as the mean ± SEM. Statistical significance was determined by 2-way ANOVA with Bonferroni post-tests (**b** and **d-middle**), 2-tailed, unpaired t test (**c** and **d-right**) and Mann–Whitney test (**e**). *P < 0.05, **P < 0.01, ***P < 0.001

**Fig. 6:**
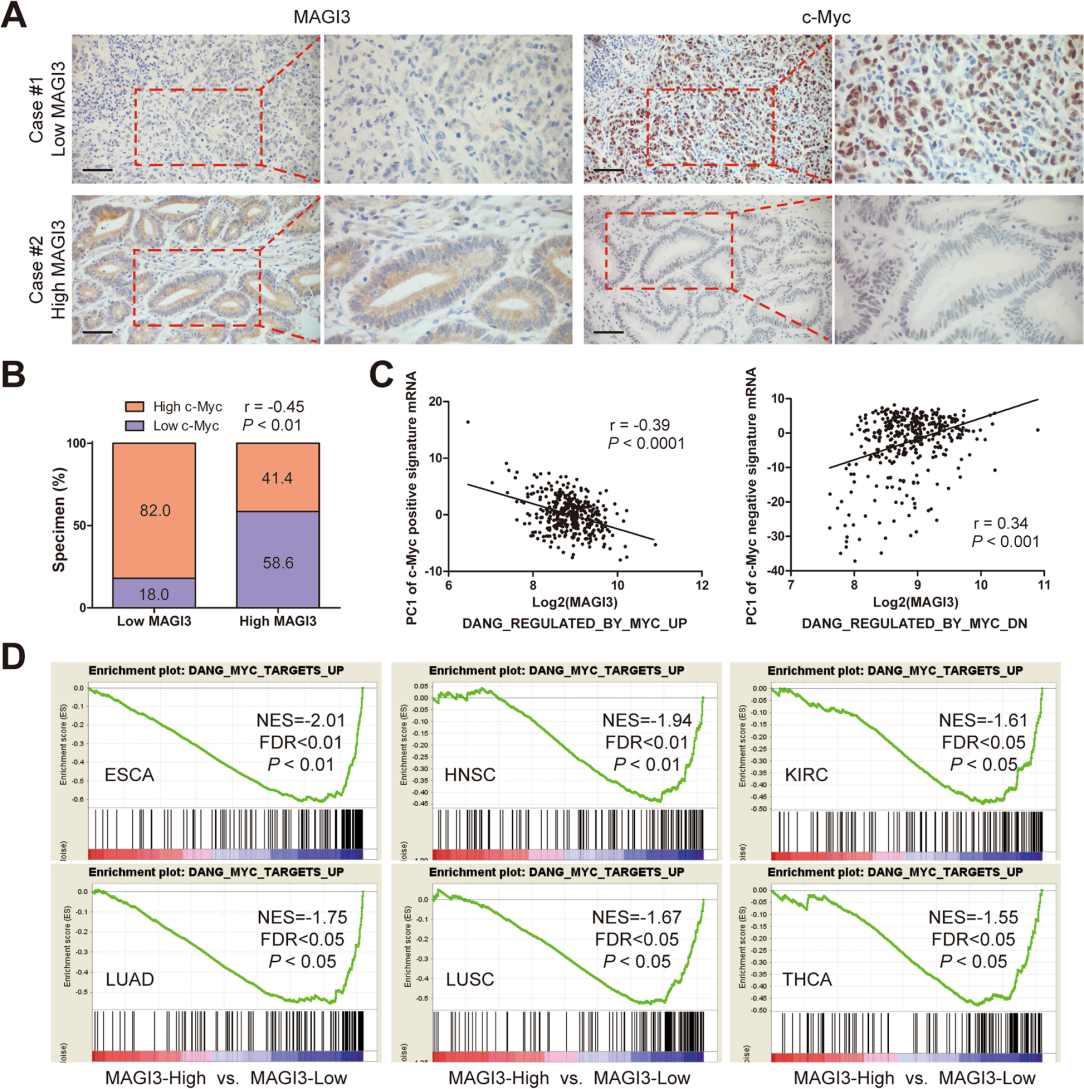
MAGI3 is negatively associated with c-Myc in CRC specimens and multiple carcinomas. **a**, The negative correlation of MAGI3 and c-Myc protein levels in clinical CRC specimens. Representative IHC staining of MAGI3 and c-Myc in 108 cases of CRC samples. Scale bars: 50 μm. Right panels are × 2 magnification of the dashed areas on the left. **b**, Pearson correlation analysis between MAGI3 and c-Myc in 108 cases of CRC samples. **c**, MAGI3 level negatively correlated with c-Myc activation in CRC patients. MAGI3 expression level was measured by RNA-Seq in 375 CRC samples from TCGA. Gene signature of c-Myc activation was defined as gene sets, DANG REGULATED BY MYC UP (the gene signature of c-Myc positive activation) and DANG REGULATED BY MYC DN (the gene signature of c-Myc negative activation) from the Molecular Signatures Database. The First Principal Component (PC1) of the c-Myc positive signature was negatively correlated with MAGI3 expression (left); while the PC1 of c-Myc negative signature was positively correlated with MAGI3 expression (right) (Pearson correlation). **d**, Enrichment plots of GSEA showed that the gene signatures of c-Myc positive activation were significantly enriched in low MAGI3 expression groups in esophageal squamous cell carcinoma (ESCA), head and neck squamous cell carcinoma (HNSC), kidney renal clear cell carcinoma (KIRC), lung adenocarcinoma (LUAD), lung squamous cell carcinoma (LUSC) and thyroid carcinoma (THCA)

To delineate whether MAGI3 suppresses CRC cell proliferation by targeting c-Myc, cell proliferation was detected in CRC-MAGI3 cells in presence or absence of c-Myc overexpression. Data showed that overexpression of c-Myc significantly promoted proliferation of HT115 or RKO cells. Overexpression of MAGI3 inhibited proliferation of HT115 or RKO cells, and downregulated c-Myc protein levels, which were consistent with our previous results. However, when c-Myc levels were overexpressed by transfection of c-Myc plasmid (Fig. 5a), the inhibition abilities of MAGI3 to CRC cells proliferation or colony formation were greatly reduced (Fig. 5b and c). Additionally, MAGI3 levels were not affected following overexpression of c-Myc in CRC cells (Additional file 4: Fig. S4A). In vivo animal experiment, MAGI3 overexpression inhibited the growth of CRC xenografts in nude mice within 15 days (Fig. 5d), and MAGI3 overexpression correlated with the reduced c-Myc expression (Fig. 5e). In AOM/DSS induced CRC of C57BL/6 mice, the expression of MAGI3 was robustly reduced coordinately with c-Myc increase compared with matched surrounding tissues (Fig. 5f). Furthermore, in our modified model of AOM/DSS induced CRC, *MAGI3*^−/−^ mice showed higher disease activity index and a significant higher tumor number and load compared to their wild-type controls (Additional file 4: Fig. S4B-E). In clinical CRC specimens, levels of MAGI3 protein were negatively correlated with expression levels of c-Myc (*r* = -0.45, Fig. 6a and b). Consistently, mRNA level of *MAGI3* was negatively correlated with the first principal component of c-Myc signaling activation (Fig. 6c). Taken together, these data indicate that MAGI3 inhibits CRC cells proliferation and CRC development through suppression of c-Myc expression and activity. Moreover, besides CRC, loss of MAGI3 expression correlated with activation of c-Myc downstream effectors in multiple carcinomas (Fig. 6d and Additional file 4: Fig. S5).

### MAGI3 regulates sensitivity of chemotherapy by inhibition of c-Myc signaling and acts as a predictor for chemotherapy response in CRC

**Fig. 7:**
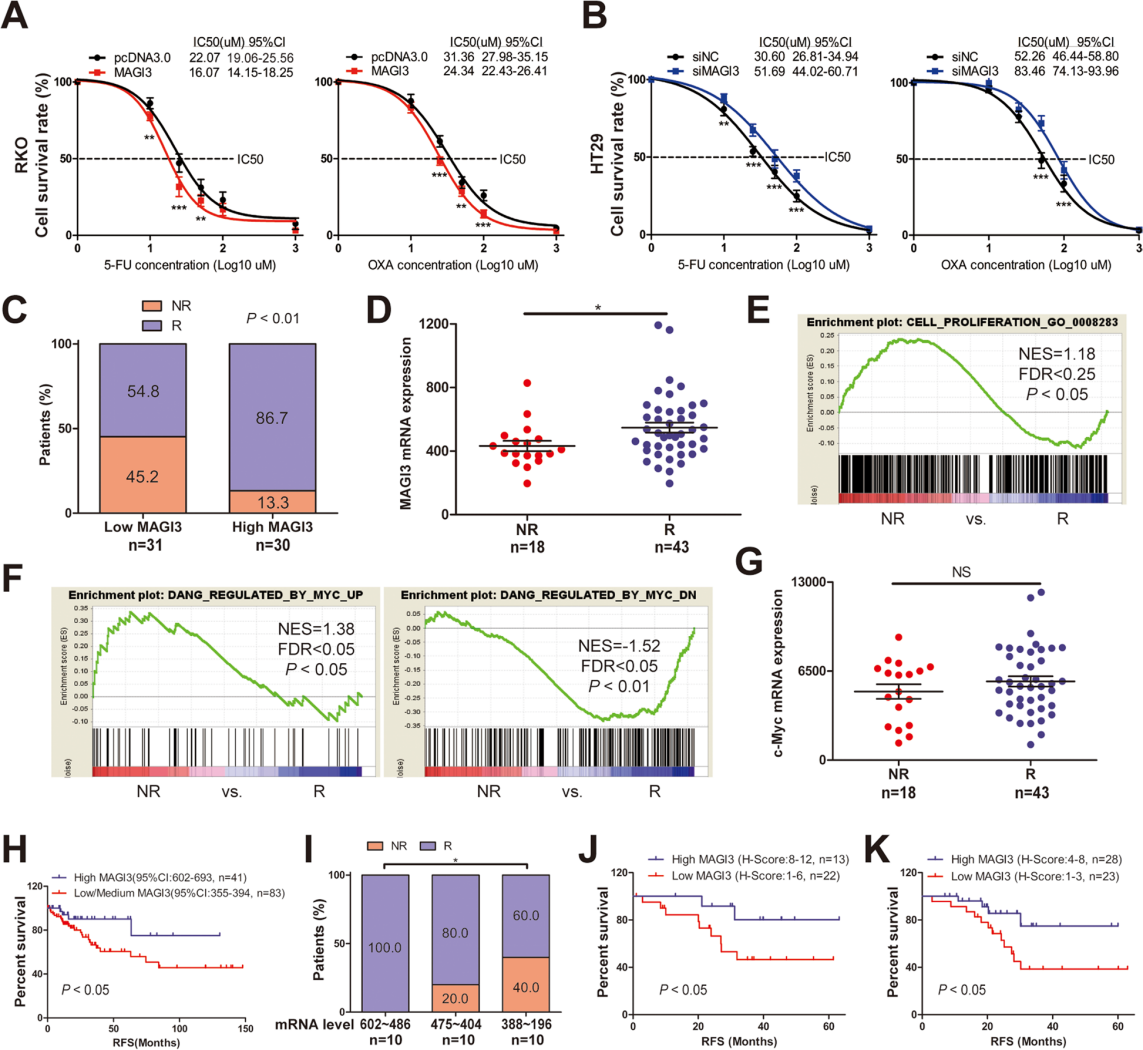
Loss of MAGI3 reduces sensitivity of chemotherapy by upregulation of c-Myc activation in CRC patients. **a** and **b**, Overexpression of MAGI3 increased, whereas knockdown of MAGI3 reduced CRC cells chemosensitivity to 5-FU or OXA. CRC cells with MAGI3 overexpressed (**a**) or knocked down (**b**) were treated with 5-FU or OXA in different concentrations for 48 h, and the cells viability were measured by CCK-8 assays (three independent experiments). **c**, The result of chi-square test showed that the patients with high *MAGI3* mRNA level had high response ratio to chemotherapy. **d**, Scatter plots of MAGI3 mRNA expression in patients with NR and R. **e** and **f**, Enrichment plots of GSEA showed that the gene signatures of cell proliferation (**e**), c-Myc positive activation (**f**, left) were enriched in NR group and c-Myc negative regulated pathway (**f**, right) was enriched in R group. **g,** Scatter plots of *c-Myc* mRNA expression in patients with NR and R. **h**, KM survival curve indicated that among the patients from TCGA without adjuvant chemotherapy, those with high *MAGI3* mRNA level had a better RFS than those with medium/low MAGI3 (log-rank test). **i**, The fisher exact test showed that among the patients receiving adjuvant chemotherapy, those with relatively medium *MAGI3* mRNA level (602 ~ 486) had significantly high response rate to fluoropyrimidine-based chemotherapy than those with relatively low *MAGI3* (388 ~ 196). **j**, KM survival curve showed that among the patients from Shanxi Medical University without adjuvant chemotherapy, those with high MAGI3 protein level (H-score ≥ 8) had a better RFS than those with medium/low MAGI3 (H-score < 8). **k**, KM survival curve showed that among the patients receiving adjuvant chemotherapy, those with medium MAGI3 protein (H-score 4 ~ 8) had a better RFS than the patients with low MAGI3 (H-score 1 ~ 3). Data were presented as the mean ± SEM, 2-way repeated-measures ANOVA with Bonferroni post-test (**a** and **b**), the chi-square test (**c**), nonparametric Mann–Whitney test (**d** and **g**), fisher exact test with 1-sided (**i**) were used to determine statistical significance. *P < 0.05, **P < 0.01, ***P < 0.001 and NS, no significance

It is well known that a high level of c-Myc is closely correlated with chemotherapy resistance in many cancers, including CRC cell [34–36], reminding that dysregulated low level of MAGI3 in CRC may reduce chemosensitivity through accumulation of c-Myc. In this study, we found that overexpression of MAGI3 significantly enhanced sensitivity of RKO cell to 5-FU or oxaliplatin (OXA) (Fig. 7a). Conversely, knockdown of MAGI3 robustly decreased sensitivity of HT29 cells to 5-FU or OXA (Fig. 7b). In clinical, a significant high ratio of response (R, complete response or partial response) to fluoropyrimidine-based chemotherapy was observed in the CRC patients with *MAGI3*-high than those of *MAGI3*-low at mRNA level (86.7% vs. 54.8%, Fig. 7c), indicating MAGI3 correlated with chemotherapy response both in vitro and in vivo. Additionally, the patients with response to chemotherapy had significantly high level of *MAGI3* than that of patients with non-response (NR, stable disease and progressive disease) (Fig. 7d), and the gene signature of cell proliferation and c-Myc activation were enriched in the non-response group (Fig. 7e and f) although mRNA level of *c-Myc* had no statistical difference between two groups (Fig. 7g). These results were further validated with an independent cohort (Additional file 4: Fig. S6). Thus, these findings provide first lines of evidences that loss of MAGI3 expression in CRC patients activates c-Myc signaling, and contributes to chemotherapy resistance in CRC.

MAGI3 as a predictive biomarker for adjuvant chemotherapy in CRC patients was further explored in this study. Clinical specimens of CRC patients in stage II/III without adjuvant chemotherapy from the TCGA were stratified into two groups by *MAGI3* mRNA level with *MAGI3*-high (95%CI, 602 ~ 693) and *MAGI3*-medium/low (95%CI, 355 ~ 394). KM survival analysis revealed that the patients of *MAGI3*-high had a significantly better RFS than the patients of *MAGI3*-medium/low (75% vs. 46% with 10-year RFS, Fig. 7h), suggesting that patients of *MAGI3*-high were not necessary for further adjuvant chemotherapy to avoid overtreatment. Subsequently, the patients of non-high *MAGI3* (mRNA level ≤ 602), who underwent adjuvant chemotherapy, were explored their chemosensitivity to fluoropyrimidine-based systemic chemotherapy. These patients were stratified into three groups by *MAGI3* mRNA level (602 ~ 486, 475 ~ 404, and 388 ~ 196), and response rates to fluoropyrimidine-based chemotherapy were 100%, 80% and 60%, respectively (Fig. 7i). These results were further validated in an independent Chinese cohort with information of RFS and MAGI3 protein level. Consistently, among the patients in stage II/III without adjuvant chemotherapy, those of MAGI3-high (MAGI3 protein level H-score ≥ 8) had a significantly better RFS than those of MAGI3-non-high (H-score < 8) (80% vs. 47% with 5-year RFS, Fig. 7j). Furthermore, among the patients receiving fluoropyrimidine-based systemic adjuvant chemotherapy, those of MAGI3-medium (H-score 4 ~ 8) had a significantly better RFS than those of MAGI3-low (H-score 1 ~ 3) (75% vs. 39% with 5-year RFS, Fig. 7k). Therefore, the patients with high MAGI3 are not necessary for further adjuvant chemotherapy to avoid overtreatment. The patients with MAGI3 in medium level are subsequently recommended to undergo fluoropyrimidine-based systemic adjuvant chemotherapy. Whereas, the patients with MAGI3 in low level may need comprehensive treatment including radiotherapy or targeted therapy. Taken together, these data indicate that MAGI3 is a potential independent predictive biomarker for chemotherapy in CRC patients.

## Discussion

Pathological staging is the major prognostic classification currently used in clinical practice to tailor patients for adjuvant chemotherapy [37, 38], but its prediction is far from accuracy to meet clinical demands. Here, we showed that low level of MAGI3 correlated with recurrence and poor prognostic of CRC by screening and validating from multi-central cohorts over a thousand cases. Low MAGI3 was identified as a risk factor of poor survival, and a predictor of CRC recurrence independent on pathological features, as well as MSI-H, *KRAS* and *BRAF* mutations. Understanding of the mechanisms by which CRC downregulates MAGI3 expression may provide new therapeutic targets for treatment of this disease. Norén et al. reported that in inflammatory bowel disease, which can further develop into CRC, inflammation was associated with decreased levels of MAGI3 [39]. In addition, based on the analysis of TCGA database, we found that methylation of MAGI3 promoter and miRNA regulation may also be the cause of down-regulate MAGI3 expression in CRC. However, the underlying molecular mechanism need to further explored.

MAGI3 have been reported binding with PTEN and β-catenin to regulate PI3K/AKT and Wnt signaling respectively, to inhibit tumor formation and progression [40, 41]. However, no study was reported the significance of these interactions in CRC cells. Analysis of TCGA database with GSEA found that gene signatures of PI3K/AKT and Wnt signaling activation were not enriched with *MAGI3* level (data not shown), reminding that MAGI3 was not involved in PI3K/AKT or Wnt signaling activation in CRC cells. Recently, Yun and his colleagues reported that MAGI3 by interaction with LPA2 and phospholipase C-β3, could decrease the tumorigenic capacity via inhibition of LPA-induced activation of NF-κB and JNK in colon cancer cells [42]. However, this conclusion was also not supported by the data from TCGA (data not shown). Given that CRC is a heterogeneous disease with genetic profiles and clinical outcomes associate with the anatomic location of the primary tumor, these results could not exclude the possibility of MAGI3/LPA2 interaction in some distinct types of CRC. Nevertheless, it is unlikely the major function of MAGI3 in CRC cells.

In this study, MAGI3 is found to promote c-Myc protein ubiquitin–proteasome degradation, subsequently attenuates c-Myc activation in a broad range of cancers besides CRC. Studies in CRC cells reveal that MAGI3 expression inhibits cell proliferation and increase cell apoptosis by downregulation of c-Myc oncogenic activities. These results are further confirmed by our in vivo studies of subcutaneous xenograft tumor, AOM/DSS induced CRC model and clinical specimens. Thereby, these findings demonstrate that MAGI3 plays an important role in CRC progression.

c-Myc is an unstable protein with a half-life of less than 30 min in non-transformed cells due to rapid turnover through the ubiquitin–proteasome system, and the SCF are the most abundant E3 ligases contribute to c-Myc degradation. Typically, F-box proteins such as FBXW7, SKP2 and STUB1, are the substrate-recognition components of SCF E3 ligase to target c-Myc [31–33]. Interestingly, our studies show that none of these F-box proteins is indispensable in regulation of c-Myc ubiquitination and degradation under the MAGI3 overexpressed condition. However, this did not suggest that MAGI3 have stronger effect than F-box proteins on the ubiquitination and degradation of c-Myc, as MAGI3 is also not indispensable in regulation of c-Myc ubiquitination and degradation when FBXW7 is overexpressed (data not shown). Knockdown either of MAGI3 or FBXW7 does not significantly alter the ubiquitination and degradation of c-Myc when the other one overexpressed. It is likely that MAGI3 and F-box proteins have function in partial redundancy in c-Myc degradation. In this study, MAGI3 is identified as a substrate-binding subunit assembled with SKP1 via a novel interface between SKP1 and the second PDZ domain of MAGI3 to form a macromolecular complex of E3 ligases MAGI3-SKP1-CUL1. MAGI3 subsequently binds c-Myc through PBM of c-Myc at its carboxyl terminal and the fifth PDZ domain of MAGI3 to associate c-Myc with the SKP1-Cullin E3 ligase, and modulates c-Myc poly-ubiquitination and degradation. To the best of our knowledge, this is the first report that MAGI3 acts as a substrate-specific binding protein of E3 to recognize c-Myc, and PDZ domain of MAGI3 is a novel domain to adapt the SKP1-Cullin E3 ligase targeting c-Myc.

Another interesting point find from this study is that MAGI3 plays an essential role in CRC chemosensitivity and acts as a potential predictor of adjuvant chemotherapy response in CRC patients. Overexpression of MAGI3 significantly enhances chemosensitivity, whereas knockdown of MAGI3 decreases the chemosensitivity to 5-FU or OXA. Analysis of chemosensitivity of CRC patients in three independent cohorts over 213 cases reveals that high MAGI3 levels are associated with good response to fluoropyrimidine-based chemotherapy. Loss of MAGI3 expression in CRC patients activate c-Myc signaling, contribute to chemotherapy resistance in CRC. Lines of evidences report that c-Myc overexpression increase chemoresistance attributing to a highly efficient DNA double stranded breaks repair system offsetting the genomic instability induced by chemotherapy, or suppression of pro-apoptotic machinery. In this study, we find that MAGI3 overexpression promotes cell apoptosis, whereas knockdown of MAGI3 suppresses cell apoptosis. Also, our GSEA results show that the gene signatures of DNA double stranded breaks repair are enriched in CRC patients with low *MAGI3* (data not shown). These results again indicate that MAGI3 regulates chemosensitivity of CRC cells via c-Myc.

In this study, we also investigate MAGI3 as a predictor for fluoropyrimidine-based adjuvant chemotherapy. The data reveal that among the stage II/III CRC patients from two independent cohorts (TCGA and a Chinese cohort) without adjuvant chemotherapy, the patients with high MAGI3 levels have significantly good RFS (~ 80% with 5-year RFS) at both mRNA and protein levels. The patients with MAGI3-medium levels (mRNA level 486 ~ 602, or protein level H-score 4 ~ 8) have significantly good response rate (100%) or RFS (80% with 5-year RFS) with fluoropyrimidine-based chemotherapy. Thereby, the patients with high MAGI3 (mRNA level > 602, protein level H-score > 8) are not necessary for further adjuvant chemotherapy; the patients with medium MAGI3 (mRNA level 486 ~ 602, protein level H-score 4 ~ 8) are subsequently recommended to undergo fluoropyrimidine-based adjuvant chemotherapy; the patients with low MAGI3 (mRNA level < 486, protein level H-score < 4) need comprehensive treatment including radiotherapy or targeted therapy. Taken together, all these results suggest that MAGI3 is a novel predictor marker to tailor adjuvant chemotherapy to improve outcome of CRC patients.

A limitation of this study is inadequately sized cohorts of CRC patients to study MAGI3 as a potential predictor for response to fluoropyrimidine-based chemotherapy, and especially in the absence of a prospectively assessed cohort. Thus, these conclusions need to be further confirmed in multiple studies with larger-scale validation across different populations.

In summary, the present study has identified MAGI3 as a novel tumor suppressor in CRC and demonstrated its inhibition on proliferation by targeting c-Myc. We provide the convincing evidence for the first time that MAGI3 is a novel E3 ubiquitin ligase by degradation of c-Myc to regulate CRC development. The loss of MAGI3 correlates with an unfavorable prognosis, and it is a potential predictive biomarker for fluoropyrimidine-based chemotherapy in CRC patients.

## Supplementary Information

**Additional file 1: Table S1.** The sequences of siRNA. **Table S2.** The gene sets of GSEA. **Table S3.** The sequence of primers. **Table S4.** The primary antibodies for western blot, CoIP and IHC. (doc 92KB)

**Additional file 2.** Supplementary materials and methods. (doc 63KB)

**Additional file 3: Table S1.** Ranking the importance of 17 differentially expressed genes. **Table S2. **Univariate and multivariate Cox regression analyses of potential poor prognostic factors in colorectal cancer. **Table S3. **Univariate and multivariate Cox regression analyses of potential poor prognostic factors in colorectal cancer from TCGA. **Table S4. **GSEA for low MAGI3 colorectal cancer patients from TCGA and GSE40967. (doc 135KB)

**Additional file 4: Figure S1.** Low MAGI3 expression is correlated with the poor prognosis of CRC patients. (A and B) Scatter plot of MAGI3 mRNA in adjacent noncancerous tissues and tumor tissues (A), non-recurrence tumors and recurrence tissues (B) from databases of TCGA and GSE40967. Data were shown as mean ± SEM, and statistical significance was calculated by 2-tailed, unpaired t test. *P<0.05, ***P<0.001. (C and D) KM survival curve showing comparison of the OS and RFS between MAGI3-low and -high expression groups of CRC patients from TCGA (C) and GSE40967 (D) databases. *P*<0.05, calculated by log-rank test. (E) Scatter plots of MAGI3 mRNA expression in MSI-H and MSS patients from TCGA. (F) Scatter plots of MAGI3 mRNA expression in KRAS mutation and KRAS mutation-free patients from TCGA. (G) Scatter plots of MAGI3 mRNA expression in BRAF abnormal and BRAF normal patients from TCGA. Data were presented as the mean ± SEM, 2-tailed, unpaired t test (E and F), nonparametric Mann-Whitney test (G) were used to determine statistical significance. NS, no significance. **Figure S2.** MAGI3 induces cell cycle arrest and apoptosis in CRC cells. (A) Protein levels of MAGI3 in indicated CRC cell lines were analyzed by western blot. (B) Establishment and identification of CRC cell lines with stable expression of exogenous MAGI3. Overexpression of MAGI3 in HT115 and RKO cells were confirmed by RT-PCR and western blot analysis. (C) Knockdown of MAGI3 by small interference RNA transfection in HT29 and SW480 cells were confirmed by RT-PCR and western blot analysis. (D) MAGI3 overexpression altered cell cycle-related protein expression. (E) MAGI3 overexpression upregulated apoptosis-related proteins expression in CRC cells. **Figure S3.** MAGI3 modulates c-Myc protein stability. (A) The half-life of c-Myc protein was markedly decreased with MAGI3 overexpression in HT115 cells. (B) The half-life of c-Myc protein was robustly increased by knockdown of MAGI3 in SW480 cells. The half-life curves of c-Myc protein were obtained by quantifying three independent experiments with western blot (right). Data were presented as the mean ± SEM, a 2-tailed, unpaired t test was used to determine statistical significance *P<0.05, **P<0.01, ***P<0.001. **Figure S4.** Knockout of MAGI3 in mice promotes the development of inflammatory CRC. (A) Overexpression of c-Myc had no detectable impacts on MAGI3 expression in HT29 and SW480 cells by western blot analysis. (B) Representative images of whole colons of WT, MAGI3^-/-^ mice at the end of the modified AOM/DSS protocol. The right image is × 2 magnification of the dashed areas on the left, in which the blue area is the mucosal surface flipped after the colon is cut off. (C) The DAI scores of MAGI3^-/-^ mice increased dramatically compared with WT mice during modified AOM/DSS protocol treatment. (D and E) The tumor numbers and tumor load were analyzed in WT and MAGI3^-/-^ mice at the end of modified AOM/DSS protocol. Data were presented as the mean ± SEM. Statistical significance was determined by 2-way ANOVA with Bonferroni post-tests (C), Mann-Whitney test (D) and fisher exact test (E), *P < 0.05, **P < 0.01, ***P < 0.001. **Figure S5.** MAGI3 expression is downregulated in multiple tumor tissues. Scatter plot of MAGI3 mRNA in adjacent noncancerous tissues and tumor tissues of BLCA, ESCA, HNSC, KIRC, KIRP, LUAD, LUSC, THCA and UCEC from databases of TCGA. Data were shown as mean ± SEM, and statistical significance was calculated by 2-tailed, unpaired t test. *P<0.05, **P<0.01, ***P<0.001. BLCA, bladder cancer; ESCA, esophageal squamous cell carcinoma; HNSC, head and neck squamous cell carcinoma; KIRC, kidney renal clear cell carcinoma; KIRP, kidney renal papillary cell carcinoma; LUAD, lung adenocarcinoma; LUSC, lung squamous cell carcinoma; THCA, thyroid carcinoma; UCEC, uterine corpus endometrial carcinoma. **Figure S6.** Loss of MAGI3 expression in CRC patients reduces sensitivity of chemotherapy by upregulation of c-Myc protein level and activation. (A) The chi-square test showed that the CRC patients from dataset GSE72970 with high MAGI3 mRNA level had high response ratio to chemotherapy. (B) Scatter plots of MAGI3 mRNA expression in patients with non-response (NR) or response (R). (C and D) Enrichment plots of GSEA showed that the gene signatures of cell proliferation (C), c-Myc positive activation (D, left) were enriched in NR group and c-Myc negative regulated pathway (D, right) was enriched in R group. (E) Scatter plots of c-Myc mRNA expression in patients with non-response (NR) or response (R). Data were presented as the mean ± SEM, the chi-square test (A), 2-tailed, unpaired t test (B and E) were used to determine statistical significance. *P<0.05 and NS, no significance. (PDF 1.86MB)

## Data Availability

The datasets used and/or analyzed during the current study are available from the corresponding author on reasonable request.

## Abbreviations

AOM: Azoxymethane
AUC: Area under curve
CCK-8: Cell counting kit-8
CEA: Carcinoembryonic antigen
CHX: Cyclohexamide
CI: Confidence interval
CoIP: Co-immunoprecipitation
CRC: Colorectal cancer
DAI: Disease activity index
DEGs: Differentially expressed genes
DSS: Dextran sulfate sodium
GSEA: Gene set enrichment analysis
GST: Glutathione S-transferase
H-score: Histochemistry score
IHC: Immunohistochemical;
KM: Kaplan–Meier
MAGI3: Membrane associated guanylate kinase, WW and PDZ domain containing 3
MDA: Mean decrease accuracy
MSI: Microsatellite instability
MSI-H: Microsatellite instability-high
OS: Overall survival
OXA: Oxaliplatin
PC1: First principal component
qPCR: Quantitative real-time-PCR
RFS: Recurrence-free survival
ROC: Receiver operating characteristic
RT-PCR: Reverse transcription PCR
SCF: SKP1-Cullin-F-box protein
siRNA: Small interfering RNA
TCGA: The cancer genome atlas
5-FU: 5-Fluorouracil
