# Supplementary material for: E3 ubiquitin ligase MAGI3 degrades c-Myc and acts as a predictor for chemotherapy response in colorectal cancer
Source: Mol Cancer. 2022 Jul 22;21:151. doi: 10.1186/s12943-022-01622-9 (PMC9306183; doi:10.1186/s12943-022-01622-9)
Supplement: Supplementary file 1 — Additional file 1: Table S1. The sequences of siRNA. Table S2. The gene sets of GSEA. Table S3. The sequence of primers. Table S4. The primary antibodies for western blot, CoIP and IHC. (doc 92KB) [file 12943_2022_1622_MOESM1_ESM.doc]

Table S1. The sequences of siRNA

| siMAGI3#1 | sense 5′-GGUCCACCAUCAGGAACAAACUCAG-3′ |
| --- | --- |
|  | anti-sense 5′-CUGAGUUUGUUCCUGAUGGUGGACC-3′ |
| siMAGI3#2 | sense 5′-GAGAUGGACCUGACCAGUCUAUAUA-3′ |
|  | anti-sense: 5′-UAUAUAGACUGGUCAGGUCCAUCUC-3′ |
| siMYC#1 | sense: 5′-GGAACAAGAAGAUGAGGAATT-3′ |
|  | anti-sense, 5′-UUCCUCAUCUUCUUGUUCCTT-3′ |
| siMYC#2 | sense 5′-ACACAAACUUGAACAGCUATT-3′ |
|  | antisense: 5′-UAGCUGUUCAAGUUUGUGUTT-3′ |
| siSKP1#1 | sense 5′-CUGAAGAUGAUGAGAACAATT-3′ |
|  | anti-sense: 5′-UUGUUCUCAUCAUCUUCAGTT-3′ |
| siSKP1#2 | sense 5′-GAGUUCUGAUGGAGAGAUATT-3′ |
|  | anti-sense 5′-UAUCUCUCCAUCAGAACUCTT-3′ |
| siFBXW7#1 | sense 5′-CCAUGCAAAGUCUCAGAAUTT-3′ |
|  | anti-sense 5′-AUUCUGAGACUUUGCAUGGTT-3′ |
| siFBXW7#2 | sense 5′-GCACUCUAUGUGCUUUCAUTT-3′ |
|  | anti-sense 5′-AUGAAAGCACAUAGAGUGCTT-3′ |
| siCHIP#1 | sense 5′-UGGAAGAGUGCCAGCGAAATT-3′ |
|  | anti-sense 5′-UUUCGCUGGCACUCUUCCATT-3′ |
| siCHIP#2 | sense 5′-GCAAGGACAUCGAGGAGCATT-3′ |
|  | anti-sense 5′-UGCUCCUCGAUGUCCUUGCTT-3′ |
| siSKP2#1 | sense 5′-CAGAAAGAAUCUCCAGAAATT-3′ |
|  | anti-sense 5′-UUUCUGGAGAUUCUUUCUGTT-3′ |
| siSKP2#2 | sense 5′-CAUCUAGACUUAAGUGAUATT-3′ |
|  | antisense: 5′-UAUCACUUAAGUCUAGAUGTT-3′ |
| control siRNA | sense 5′-UUCUCCGAACGUGUCACGUTT-3′ |
|  | antisense 5′-ACGUGACACGUUCGGAGAATT-3′ |

Table S2. The gene sets of GSEA

| SIGNATURE | NAME |
| --- | --- |
| c-Myc signatures | HALLMARK_MYC_TARGETS_V1 |
| c-Myc upregulated genes | DANG_REGULATED_BY_MYC_UP |
| c-Myc downregulated genes | DANG_REGULATED_BY_MYC_DN |
| cell proliferation | CELL_PROLIFERATION_GO_0008283 |
| DNA double strand break  repair pathway | REACTOME_DNA_DOUBLE_STRAND_BREAK_REPAIR |
|  | GO_DOUBLE_STRAND_BREK_REPAIR |
| AKT pathway | HALLMARK_PI3K_AKT_MTOR_SIGNALING |
| WNT pathway | HALLMARK_WNT_BETA_CATENIN_SIGNALING |
| PLC pathway | BIOCARTA _PLC_PATHWAY |
| NF-KB pathway | BIO-CARTA_NFKB_PATHWAY |
| NF-KB upregulated genes | MARTIN_NFKB_TARGETS_UP |
| NF-KB downregulated genes | MARTIN_NFKB _TARGETS_DN |

Table S3. The sequence of primers

| MAGI3 | forward 5'-CCTCGGTCACACTCATGCAGATG-3 ' |
| --- | --- |
|  | reverse 5'-GGTAGCAGCAACAATGTCCACAAC-3' |
| c-Myc | forward 5'-TTGGAGCGCCAGAGGAGGAAC-3' |
|  | reverse 5'-GCTTGGACGGACAGGATGTATGC-3' |
| CCND2 | forward 5'-TTACACCGACAACTCCATCAAGCC-3' |
|  | reverse 5'-TGCCAGGTTCCACTTCAACTTCC-3' |
| CDK4 | forward 5'-CTGGTGACAAGTGGTGGAACAGTC-3' |
|  | reverse 5'-GGTGTAAGTGCCATCTGGTAGCTG-3' |
| CDC25A | forward 5'-GATGATGGCTTCGTGGACCTTCTC-3' |
|  | reverse 5'-ACTGACCGAGTGCTGGAGCTAC-3' |
| CCNA2 | forward 5'-AGCAGAGGCCGAAGACGAGAC-3' |
|  | reverse 5'-CCAAGGAGGAACGGTGACATGC-3' |
| CDKN1A | forward 5'-GCTGAGCCGCGACTGTGATG-3' |
|  | reverse 5'-CCTCCAGTGGTGTCTCGGTGAC-3' |
| CASP3 | forward 5'-TGGAAGCGAATCAATGGACTCTGG-3' |
|  | reverse 5'-CAGACCGAGATGTCATTCCAGTGC-3' |
| GADD45A | forward 5'-TGCGAGAACGACATCAACATCCTG-3' |
|  | reverse 5'-TGAATGTGGATTCGTCACCAGCAC-3' |
| PCNA | forward 5'-TCGTCCCACGTCTCTTTGGT-3' |
|  | reverse 5'-ATCTTCATTGCCGGCGCATT-3' |
| EIF4E | forward 5'-CTGCGGCTGATCTCCAAGTT-3' |
|  | reverse 5'-TGAGTAGTCACAGCCAGGCAT-3' |

Table S4. The primary antibodies for western blot, CoIP and IHC

| MAGI3 | Novus | #NBP2-17210 |
| --- | --- | --- |
| MAGI3 | Santa | sc-136471 |
| c-Myc | Abcam | ab32072 |
| SKP1 | Abcam | ab80586 |
| FBXW7 | ABclonal | A5872 |
| SKP2 | ABclonal | A4046 |
| STUB1 | ABclonal | A11751 |
| CUL1 | ABclonal | A19034 |
| CDK4 | ABclonal | A0366 |
| p21 | ABclonal | A19094 |
| PCNA | ABclonal | A12427 |
| Cyclin D1 | ABclonal | A19038 |
| Ubiquitin | CST | CST#3933 |
| Cleaved Caspase-9 | CST | CST#20750 |
| Cleaved Caspase-7 | CST | CST#8438 |
| Cleaved Caspase-3 | CST | CST#9664 |
| Cleaved PARP | CST | CST#5625 |
| Flag | CST | CST#14793 |
| His | CST | CST#12698 |
| GST | CST | CST#2622 |
| β-actin | Bioss | bs-0061R |
