# Supplementary material for: E3 ubiquitin ligase MAGI3 degrades c-Myc and acts as a predictor for chemotherapy response in colorectal cancer
Source: Mol Cancer. 2022 Jul 22;21:151. doi: 10.1186/s12943-022-01622-9 (PMC9306183; doi:10.1186/s12943-022-01622-9)
Supplement: Supplementary file 2 — Additional file 2. Supplementary materials and methods. (doc 63KB) [file 12943_2022_1622_MOESM2_ESM.doc]

**Supplementary Materials and Methods**

**DEGs screening**

The edgeR package (https://www.omicshare.com/tools/Home/Soft/diffanalysis) was applied to screen DEGs from databases of TCGA and GSE40967 between tumor vs. adjacent noncancerous tissues, and recurrence vs. nonrecurrence tumors. *P*<0.01 and fold change >1.5 were selected as the cutoff criterion for DEGs screening. The online tool Volcano Plot (https://www.omicshare.com/tools/Home/Soft/volcano) was used to make volcanic maps of genes between tumor vs. adjacent noncancerous tissues, and recurrence vs. nonrecurrence tumors. The online tool UpSetVenny (<https://www.omicshare.com/tools/Home/Soft/>seniorvenn) was used to compare the DEGs from TCGA and GSE40967 and to identify the common overlapped DEGs. The genes that presented in both TCGA and GSE40967 were selected as the final DEGs.

**Cell culture**

The human CRC cell lines HT115, RKO, HRT18, Caco2, HT29, SW480、SW620 and HEK293 cell lines were purchased from the National Infrastructure of Cell Line Resource (Beijing, China). Cells were grown in MEM medium (Gibco, Carlsbad, CA) for RKO, Caco2 and HEK293 cells, or RPMI-1640 medium for HT115 and HRT18 cells, or IMDM medium for SW480 cells, or L15 medium for SW620 cells, or DMEM/F12 medium for HT29 cells, supplemented with 10% (20% for Caco2) fetal bovine serum (complete medium) and 1% penicillin/streptomycin under a humidified atmosphere of 5% CO_2_ at 37°C. Cell culture reagents were purchased from HyClone (Logan, UT).

**Plasmid or siRNA transfection**

For transfection, cells were seeded into a 6-well plate and then transfected with 1μg plasmids or 80 nmol/L siRNA using Lipofectamine 3000 following the manufacturer's instructions. For siRNA knockdown and transient transfection experiments, cells were collected 48h post-transfection for protein extraction. For establishment of stable MAGI3-expressing cells, cells were transfected with control vector or constructs of MAGI3. After 24h transfection, the cells were selected with 800 μg/mL G418 (Calbiochem, SanDiego, CA) in culture medium for an additional 14 days. Expression of the MAGI3 mRNA and protein were verified by reverse transcription PCR and western blot analysis.

**Cell proliferation measurement**

Cells were cultured in 96-well plates (3000 cells per well). At indicated hours after plating, Cell counting kit-8 [CCK-8 (Dojindo, Kumamoto, Japan)] was added to each well according to the manufacturer's instructions and the cells were cultured for another 1h. Then, viable cells were quantified by measuring absorbance at 450nm with an EnSpire label microplate reader (PerkinElmer, Waltham, MA).

**Colony formation assay**

Cells were digested in 0.25% trypsin to reconstitute the single-cell suspension at a density of 1.0x10^5^ cells per ml. Cells were then seeded in six-well plates (500 cells per well) and incubated at 37°C and at an atmosphere of 5% CO_2_ for 10~14 days. The supernatants were discarded, and the colonies washed with PBS for twice and fixed in 4% paraformaldehyde for 20 min. Cells were further stained with crystal violet for 20 min and then recycle the crystal violet and rinse the six-well plates carefully with clear water and allowed to air dry at room temperature. The experiments were triplicated and the colonies (>50 cells) were counted.

**Cell cycle and apoptosis analysis**

Cell cycles were examined by ﬂow cytometry. After ﬁxing for 30 min in 70% cold ethanol, cells were then incubated with propidium iodide for 30 min, then analyzed by ﬂow cytometry (EPICS@XL, Beckman Coulter, 250 S. Kraemer Boulevard Brea, CA). Cell apoptosis were examined by ﬂow cytometry (BD LSRFortessa, Becton, Dickinson and Company, Franklin lake, NJ). Apoptosis detection was conducted using the Annexin V-FITC Apoptosis Detection Kit purchased from Solarbio (Beijing, China). The cells were collected using EDTA free trypsin and then resuspended in PBS. After centrifuged, the cells were then stained with 5 μl fluorescein isothiocyanate (FITC)-Annexin V, incubated at room temperature protected from light and then stained with 10 μl propidium iodide (20 μg /ml).

**Plasmid constructions and RNA interference**

The constructs encoding Flag-c-Myc-wt, Flag-c-Myc-∆ct, GST-c-Myc-wt, GST-c-Myc-∆ct, GST-Skp1, HA-Ub and HA-Ub/K48R were purchased from UNIBIO company (www.unibio.net). pcDNA3-V5/His-MAGI3 were kindly provided by Dr. Randy Hall (Emory University, GA). pET30A plasmids containing the PDZ domains of MAGI3 were generated as described previously [1]. Small interfering RNA (siRNA) duplexes were synthesized and purchased from Sangon Biotech Co., Ltd (Shanghai, China) and the siRNA sequences were listed in Additional file 1: Table S1.

**Gene set enrichment analysis (GSEA)**

GSEA was carried out as previously [2] to assess the enrichment of gene sets obtained from Molecular Signatures Database (http://software.broadinstitute.org/gsea/msigdb). The gene sets were listed in Additional file 1: Table S2. Tests were performed by using the default settings, and permutations number was set at 1000. False discovery rate <0.25 and *P*<0.05 were considered statistically signiﬁcant.

**Quantitative Real-time-PCR (qPCR) and RT-PCR**

Total RNA was extracted from cells using RAN-Quick Purification Kit (Yishan Biotechnology, #RN001, Shanghai, China) according to the manufacturer's instructions. First-strand cDNA was generated with HiScript® III RT SuperMix for qPCR (+gDNA wiper) (Vazyme, #R323, Nanjing, China). qPCR was carried out according to the manufacturer's instructions (Life Technologies). As RT-PCR, the cDNAs obtained were mixed with PCR solution containing the corresponding primers. The following PCR was cycled 35 times at 94°C for 30 s, 60°C for 45 s, and 65°C for 30 s. The PCR products were resolved by agarose gels. The gene expression was normalized against β-actin in the same sample. The primers used were listed in Additional file 1: Table S3.

**Western blot analysis and reagent**

Western blot was performed as described previously [3]. The primary antibodies used were listed in Additional file 1: Table S4. The immune complex was detected using horse radish peroxidase (HRP)-conjugated secondary antibodies (ZSGB-BIO, Beijing, China). Anti-mouse and anti-rabbit secondary antibodies were purchased from Amersham Biosciences (Piscataway, NJ). Immunoreactive bands were quantiﬁed by NIH Image 1.62 (National Institutes of Health, Bethesda MD). MG132 (HY-13259) was purchased from MedChemExpress (Shanghai, China); Cycloheximide (GC17198) was purchased from GLPBIO (Montclair, CA).

**IHC analysis**

The xenograft tumors and CRC samples tissues were fixed with 4% formalin and prepared as paraffin-embedded section. The tissue slides were deparaffinized in xylene and rehydrated through graded alcohol baths. Sections were either stained with hematoxylin and eosin (KeyGen Biotech) for morphological evaluation, or antigen retrieval for IHC studies. For antigen retrieval methods, slides were submerged into EDTA antigenic retrieval buffer and microwaved, followed by treatment with 3% hydrogen peroxide in methanol to quench the endogenous peroxidase activity and incubation with 3% bovine serum albumin to block the nonspecific binding. Anti-MAGI3 (1:100; sc-136471, Santa Cruz, Dallas, TX), anti-c-Myc (1:100; ab32072, Abcam) antibody were incubated with the tissues overnight at 4°C followed by incubation with horseradish-peroxidase (HRP)-conjugated secondary antibody kits. Afterwards, the expression of MAGI3 and c-Myc in tissue slides were assessed by two pathologists who were blinded to clinical and pathologic information. The staining outcomes were assessed as the intensity on a scale of 0 to 3 as no staining, weak staining, moderate staining, strong staining, and semi-quantitative assessment of positive tumor cell percentage, which was on a scale of 0 to 4 as 0 (none), 1 (1–25%), 2 (26–50%), 3 (51–75%), or 4 (>75%). H-score of staining was calculated by multiplying these two variables.

**Supplementary References**

1. He J, Bellini M, Inuzuka H, Xu J, Xiong Y, Yang X, Castleberry AM, Hall RA. Proteomic Analysis of β1-Adrenergic Receptor Interactions with PDZ Scaffold Proteins. J BIOL CHEM 2006; 281: 2820-7.

2. Wang Q, Qin Q, Song R, Zhao C, Liu H, Yang Y, Gu S, Zhou D, He J. NHERF1 inhibits beta-catenin-mediated proliferation of cervical cancer cells through suppression of alpha-actinin-4 expression. CELL DEATH DIS 2018; 9

3. Tao T, Yang X, Zheng J, Feng D, Qin Q, Shi X, Wang Q, Zhao C, Peng Z, Liu H, et al. PDZK1 inhibits the development and progression of renal cell carcinoma by suppression of SHP-1 phosphorylation. ONCOGENE 2017; 36: 6119-31.
