# Supplementary material for: E3 ubiquitin ligase MAGI3 degrades c-Myc and acts as a predictor for chemotherapy response in colorectal cancer
Source: Mol Cancer. 2022 Jul 22;21:151. doi: 10.1186/s12943-022-01622-9 (PMC9306183; doi:10.1186/s12943-022-01622-9)
Supplement: Supplementary file 3 — Additional file 3: Table S1. Ranking the importance of 17 differentially expressed genes. Table S2. Univariate and multivariate Cox regression analyses of potential poor prognostic factors in colorectal cancer. Table S3. Univariate and multivariate Cox regression analyses of potential poor prognostic factors in colorectal cancer from TCGA. Table S4. GSEA for low MAGI3 colorectal cancer patients from TCGA and GSE40967. (doc 135KB) [file 12943_2022_1622_MOESM3_ESM.doc]

Supplementary Table 1. Ranking the importance of 17 differentially expressed genes

^a^, TCGA (Normal vs. Adenocarcinoma); ^b^, TCGA (Recurrence-free vs. Recurrence);

| Gene | Mean Decrease Accuracy (importance rank) | | | | Sum of rank |
| --- | --- | --- | --- | --- | --- |
|  | TCGA^a^ | TCGA^b^ | GSE40967^c^ | GSE40967^d^ |  |
| MAGI3 | 34.5(4) | 16.3(2) | 36.9(1) | 16.8(3) | 10 |
| SFTA2 | 41.9(2) | 13.0(3) | 25.6(5) | 10.5(7) | 17 |
| STMN2 | 64.0(1) | 2.2(14) | 31.8(2) | 22.2(1) | 18 |
| LEMD1 | 39.5(3) | 10.1(7) | 19.1(9) | 12.9(6) | 25 |
| SLC35G1 | 17.8(11) | 2.3(13) | 27.5(4) | 21.0(2) | 30 |
| HEPACAM2 | 29.1(6) | 10.3(6) | 31.0(3) | -0.3(17) | 32 |
| NKD1 | 27.7(7) | 12.6(4) | 18.7(10) | 6.3(12) | 33 |
| COL9A3 | 15.9(12) | 5.2(10) | 23.3(8) | 13.5(5) | 35 |
| GRP | 3.8(17) | 28.9(1) | 24.2(6) | 6.9(11) | 35 |
| CLDN11 | 31.7(5) | 12.1(5) | 12.8(14) | 0.4(16) | 40 |
| IL1R2 | 17.9(10) | -0.9(17) | 17.8(11) | 15.9(4) | 42 |
| AKR1C1 | 10.5(15) | 5.9(8) | 16.0(13) | 9.3(8) | 44 |
| NXPE1 | 19.4(9) | 2.1(15) | 24.0(7) | 4.1(14) | 45 |
| ST6GALNAC1 | 14.2(13) | 5.1(11) | 16.7(12) | 7.0(10) | 46 |
| REG3A | 10.6(14) | 5.2(9) | 10.4(15) | 7.4(9) | 47 |
| CDC6 | 25.8(8) | 1.3(16) | 6.8(17) | 4.3(13) | 54 |
| IFI6 | 9.7(16) | 4.9(12) | 7.1(16) | 2.5(15) | 59 |

^c^, GSE40967 (Normal vs. Adenocarcinoma); ^d^, GSE40967 (Recurrence-free vs. Recurrence).

Supplementary Table 2. Univariate and multivariate Cox regression analyses of potential poor prognostic factors in colorectal cancer

|  | Univariate |  |  | Multivariate |  |
| --- | --- | --- | --- | --- | --- |
| Variable | RR(95%CI) | *P* Value |  | RR(95%CI) | *P* Value |
| Age(year) |  |  |  |  |  |
| >60 | 1.282 (0.615 to 2.67) | 0.508 |  | 0.985 (0.445 to 2.183) | 0.971 |
| ≤60 | 1 |  |  | 1 |  |
| Gender |  |  |  |  |  |
| Male | 0.755 (0.357 to 1.595) | 0.461 |  | 0.950 (0.0413 to 2.184) | 0.903 |
| Female | 1 |  |  | 1 |  |
| Differentiation |  |  |  |  |  |
| Moderate or high | 1.139 (0.532 to 2.438) | 0.738 |  |  |  |
| Low | 1 |  |  |  |  |
| TNM stage |  |  |  |  |  |
| Ⅰ | 0.039 (0.008 to 0.200) | < 0.001 |  | 0.094 (0.014 to 0.613) | 0.014 |
| Ⅱ | 0.079 (0.019 to 0.329) | < 0.001 |  | 0.175 (0.036 to 0.852) | 0.031 |
| Ⅲ | 0.132 (0.034 to 0.515) | 0.004 |  | 0.172 (0.040 to 0.737) | 0.018 |
| Ⅳ | 1 |  |  | 1 |  |
| MAGI3 H-score |  |  |  |  |  |
| Low | 3.592 (1.682 to 7.673) | 0.001 |  | 3.017 (1.170 to 7.778) | 0.022 |
| High | 1 |  |  | 1 |  |

RR, relative risk; TNM, Tumor-Nodes-Metastasis.

Supplementary Table 3. Univariate and multivariate Cox regression analyses of potential poor prognostic factors in colorectal cancer from TCGA

|  | Univariate |  |  | Multivariate |  |  |
| --- | --- | --- | --- | --- | --- | --- |
| Variable | RR(95%CI) | P Value |  | RR(95%CI) | P Value |  |
| Age(year) |  |  |  |  |  |  |
| ≤60 | 1.667 (0.829 to 3.353) | 0.151 |  | 1.871 (0.875 to 4.002) | 0.106 |  |
| >60 | 1 |  |  | 1 |  |  |
| Gender |  |  |  |  |  |  |
| Male | 1.516 (0.798 to 2.880) | 0.204 |  | 1.467 (0.734 to 2.934) | 0.278 |  |
| Female | 1 |  |  | 1 |  |  |
| Vascular Invasion |  |  |  |  |  |  |
| YES | 2.141 (1.135 to 4.039) | 0.019 |  | 1.771 (0.624 to 5.025) | 0.283 |  |
| NO | 1 |  |  |  |  |  |
| Lymphatic Vessel Invasion |  |  |  |  |  |  |
| YES | 2.076 (1.099 to 3.922) | 0.024 |  | 0.727 (0.242 to 2.188) | 0.571 |  |
| NO | 1 |  |  |  |  |  |
| Perineural Invasion |  |  |  |  |  |  |
| YES | 1.597 (0.818 to 3.115) | 0.170 |  |  |  |  |
| NO | 1 |  |  |  |  |  |
| TNM stage |  |  |  |  |  |  |
| Ⅰ | 0.178 (0.057 to 0.549) | 0.003 |  | 0.173 (0.051 to 0.594) | 0.005 |  |
| Ⅱ | 0.151 (0.060 to 0.381) | < 0.001 |  | 0.142 (0.051 to 0.392) | < 0.001 |  |
| Ⅲ | 0.392 (0.186 to 0.829) | 0.014 |  | 0.424 (0.193 to 0.932) | 0.033 |  |
| Ⅳ | 1 |  |  | 1 |  |  |
| MAGI3 mRNA level |  |  |  |  |  |  |
| Low | 2.270 (1.177 to 4.378) | 0.014 |  | 2.121 (1.088 to 4.135) | 0.027 |  |
| High | 1 |  |  | 1 |  |  |

RR, relative risk; TNM, Tumor-Nodes-Metastasis.

| TCGA | | | |  | GSE40967 | | | |
| --- | --- | --- | --- | --- | --- | --- | --- | --- |
| GENE SET NAME | NES | P | FDR |  | GENE SET NAME | NES | P | FDR |
| HALLMARK_MYC_TARGETS_V1 | -2.58 | 0 | 0 |  | HALLMARK_E2F_TARGETS | -3.24 | 0 | 0 |
| HALLMARK_MYC_TARGETS_V2 | -2.45 | 0 | 0 |  | HALLMARK_MYC_TARGETS_V1 | -3.16 | 0 | 0 |
| HALLMARK_EPITHELIAL_MESENCHYMAL_TRANSITION | -2.3 | 0 | 0 |  | HALLMARK_MYC_TARGETS_V2 | -2.87 | 0 | 0 |
| HALLMARK_OXIDATIVE_PHOSPHORYLATION | -2.29 | 0 | 0 |  | HALLMARK_G2M_CHECKPOINT | -2.79 | 0 | 0 |
| HALLMARK_DNA_REPAIR | -2.28 | 0 | 0 |  | HALLMARK_MTORC1_SIGNALING | -2.59 | 0 | 0 |
| HALLMARK_INTERFERON_ALPHA_RESPONSE | -2.01 | 0 | 0 |  | HALLMARK_OXIDATIVE_PHOSPHORYLATION | -2.40 | 0 | 0 |
| HALLMARK_INTERFERON_GAMMA_RESPONSE | -1.99 | 0 | 0 |  | HALLMARK_UNFOLDED_PROTEIN_RESPONSE | -2.22 | 0 | 0 |
| HALLMARK_UNFOLDED_PROTEIN_RESPONSE | -1.81 | 0 | 0.0006 |  | HALLMARK_DNA_REPAIR | -2.13 | 0 | 0 |
| HALLMARK_MTORC1_SIGNALING | -1.80 | 0 | 0.0007 |  | HALLMARK_CHOLESTEROL_HOMEOSTASIS | -1.84 | 0 | 0.0004 |
| HALLMARK_E2F_TARGETS | -1.75 | 0 | 0.0015 |  | HALLMARK_SPERMATOGENESIS | -1.74 | 0 | 0.0013 |

Supplementary Table 4. GSEA for low MAGI3 colorectal cancer patients from TCGA and GSE40967
