# Supplementary material for: E3 ubiquitin ligase MAGI3 degrades c-Myc and acts as a predictor for chemotherapy response in colorectal cancer
Source: Mol Cancer. 2022 Jul 22;21:151. doi: 10.1186/s12943-022-01622-9 (PMC9306183; doi:10.1186/s12943-022-01622-9)
Supplement: Supplementary file 4 — Additional file 4: Figure S1. Low MAGI3 expression is correlated with the poor prognosis of CRC patients. (A and B) Scatter plot of MAGI3 mRNA in adjacent noncancerous tissues and tumor tissues (A), non-recurrence tumors and recurrence tissues (B) from databases of TCGA and GSE40967. Data were shown as mean ± SEM, and statistical significance was calculated by 2-tailed, unpaired t test. *P<0.05, ***P<0.001. (C and D) KM survival curve showing comparison of the OS and RFS between MAGI3-low and -high expression groups of CRC patients from TCGA (C) and GSE40967 (D) databases. P<0.05, calculated by log-rank test. (E) Scatter plots of MAGI3 mRNA expression in MSI-H and MSS patients from TCGA. (F) Scatter plots of MAGI3 mRNA expression in KRAS mutation and KRAS mutation-free patients from TCGA. (G) Scatter plots of MAGI3 mRNA expression in BRAF abnormal and BRAF normal patients from TCGA. Data were presented as the mean ± SEM, 2-tailed, unpaired t test (E and F), nonparametric Mann-Whitney test (G) were used to determine statistical significance. NS, no significance. Figure S2. MAGI3 induces cell cycle arrest and apoptosis in CRC cells. (A) Protein levels of MAGI3 in indicated CRC cell lines were analyzed by western blot. (B) Establishment and identification of CRC cell lines with stable expression of exogenous MAGI3. Overexpression of MAGI3 in HT115 and RKO cells were confirmed by RT-PCR and western blot analysis. (C) Knockdown of MAGI3 by small interference RNA transfection in HT29 and SW480 cells were confirmed by RT-PCR and western blot analysis. (D) MAGI3 overexpression altered cell cycle-related protein expression. (E) MAGI3 overexpression upregulated apoptosis-related proteins expression in CRC cells. Figure S3. MAGI3 modulates c-Myc protein stability. (A) The half-life of c-Myc protein was markedly decreased with MAGI3 overexpression in HT115 cells. (B) The half-life of c-Myc protein was robustly increased by knockdown of MAGI3 in SW480 cells. The half-life curves [file 12943_2022_1622_MOESM4_ESM.pdf]

Supplementary Figure 1

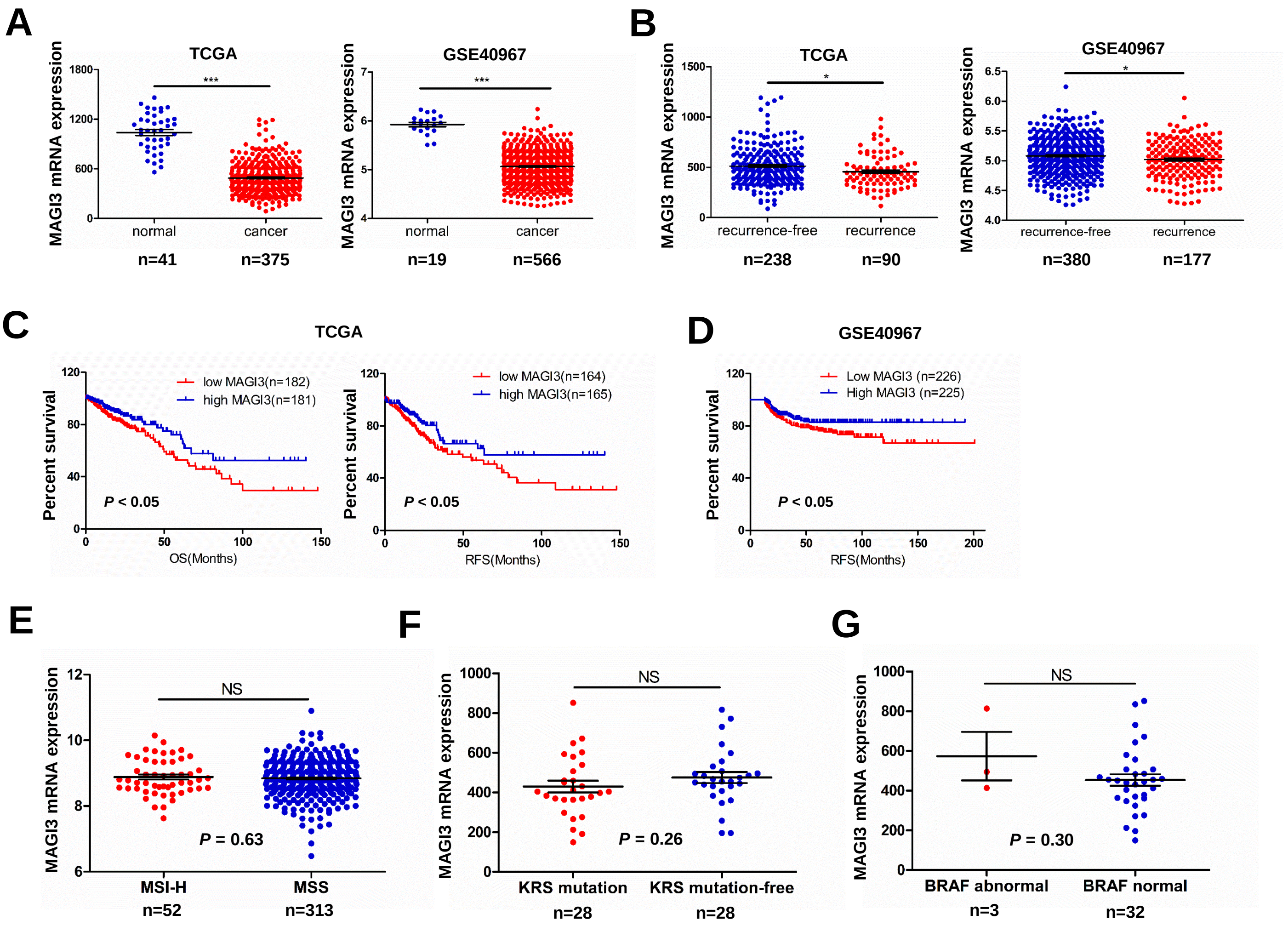

Figure S1. Low MAGI3 expression is correlated with the poor prognosis of CRC patients.
(A and B) Scatter plot of *MAGI3* mRNA in adjacent noncancerous tissues and tumor tissues (A), non-recurrence tumors and recurrence tissues (B) from databases of TCGA and GSE40967. Data were shown as mean ± SEM, and statistical significance was calculated by 2-tailed, unpaired t test. *P<0.05, ***P<0.001. (C and D) KM survival curve showing comparison of the OS and RFS between *MAGI3*-low and -high expression groups of CRC patients from TCGA (C) and GSE40967 (D) databases. P<0.05, calculated by log-rank test. (E) Scatter plots of MAGI3 mRNA expression in MSI-H and MSS patients from TCGA. (F) Scatter plots of MAGI3 mRNA expression in KRAS mutation and KRAS mutation-free patients from TCGA. (G) Scatter plots of MAGI3 mRNA expression in BRAF abnormal and BRAF normal patients from TCGA. Data were presented as the mean ± SEM, 2-tailed, unpaired t test (E and F), nonparametric Mann-Whitney test (G) were used to determine statistical significance. NS, no significance.

Supplementary Figure 2

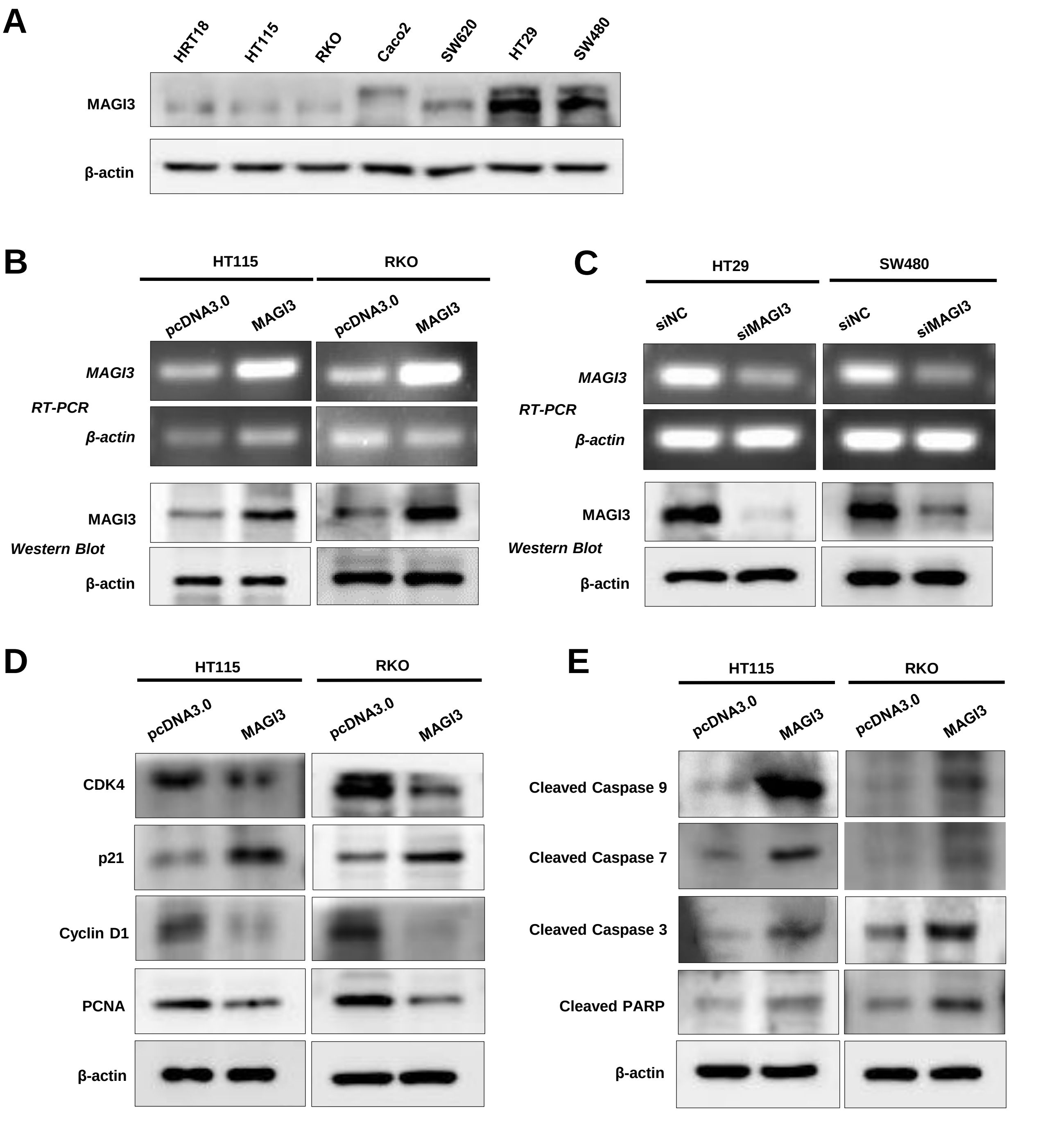

Figure S2. MAGI3 induces cell cycle arrest and apoptosis in CRC cells.
(A) Protein levels of MAGI3 in indicated CRC cell lines were analyzed by western blot. (B) Establishment and identification of CRC cell lines with stable expression of exogenous MAGI3. Overexpression of MAGI3 in HT115 and RKO cells were confirmed by RT-PCR and western blot analysis. (C) Knockdown of MAGI3 by small interference RNA transfection in HT29 and SW480 cells were confirmed by RT-PCR and western blot analysis. (D) MAGI3 overexpression altered cell cycle-related protein expression. (E) MAGI3 overexpression upregulated apoptosis-related proteins expression in CRC cells.

Supplementary Figure 3

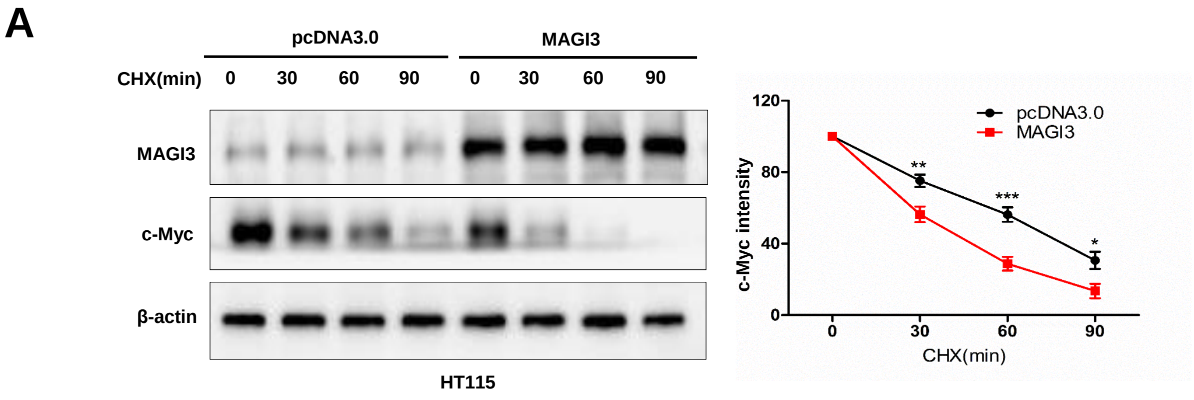

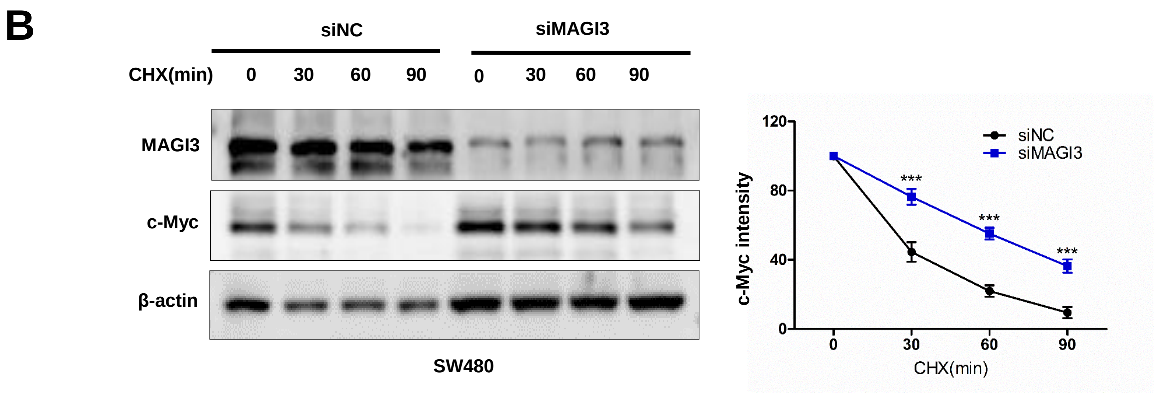

Figure S3. MAGI3 modulates c-Myc protein stability.
(A) The half-life of c-Myc protein was markedly decreased with MAGI3 overexpression in HT115 cells. (B) The half-life of c-Myc protein was robustly increased by knockdown of MAGI3 in SW480 cells. The half-life curves of c-Myc protein were obtained by quantifying three independent experiments with western blot (right). Data were presented as the mean ± SEM, a 2-tailed, unpaired t test was used to determine statistical significance *P<0.05, **P<0.01, ***P<0.001.

Supplementary Figure 4

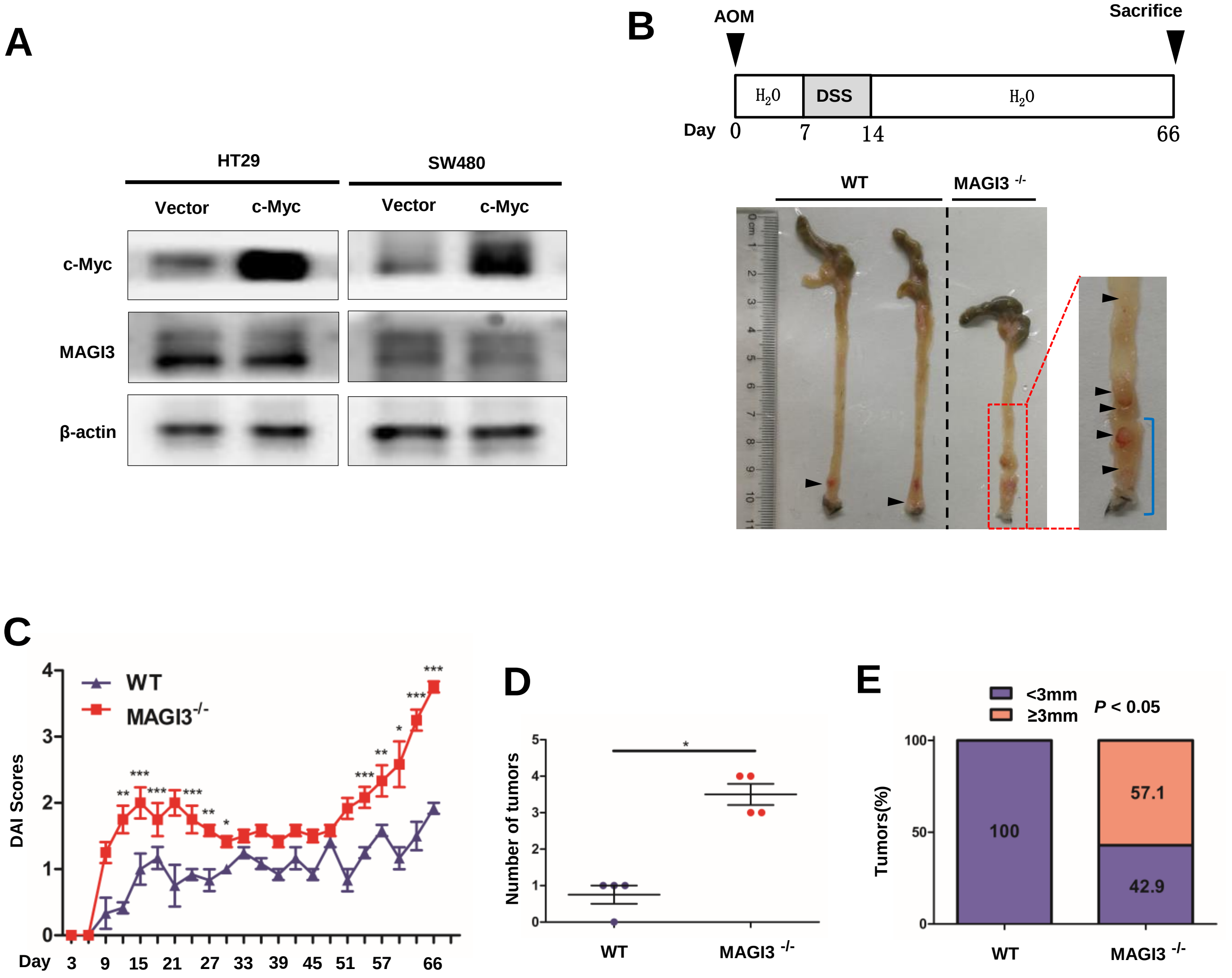

Figure S4. Knockout of MAGI3 in mice promotes the development of inflammatory CRC.
(A) Overexpression of c-Myc had no detectable impacts on MAGI3 expression in HT29 and SW480 cells by western blot analysis. (B) Representative images of whole colons of WT, MAGI3$^{-/-}$ mice at the end of the modified AOM/DSS protocol. The right image is x 2 magnification of the dashed areas on the left, in which the blue area is the mucosal surface flipped after the colon is cut off. (C) The DAI scores of MAGI3$^{-/-}$ mice increased dramatically compared with WT mice during modified AOM/DSS protocol treatment. (D and E) The tumor numbers and tumor load were analyzed in WT and MAGI3$^{-/-}$ mice at the end of modified AOM/DSS protocol. Data were presented as the mean ± SEM. Statistical significance was determined by 2-way ANOVA with Bonferroni post-tests (C), Mann-Whitney test (D) and fisher exact test (E), *P < 0.05, **P < 0.01, ***P < 0.001.

Supplementary Figure 5

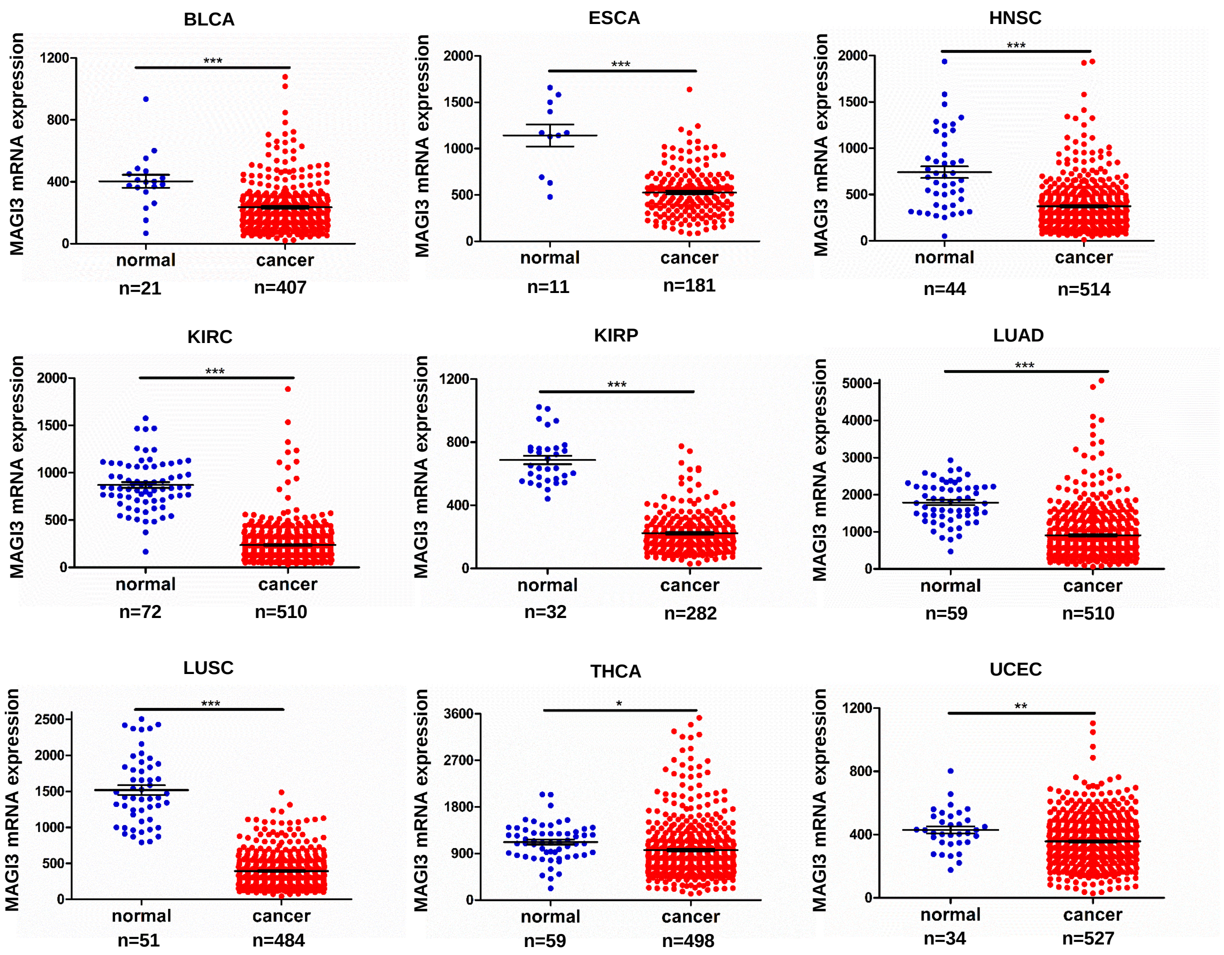

Figure S5. MAGI3 expression is downregulated in multiple tumor tissues.
Scatter plot of *MAGI3* mRNA in adjacent noncancerous tissues and tumor tissues of BLCA, ESCA, HNSC, KIRC, KIRP, LUAD, LUSC, THCA and UCEC from databases of TCGA. Data were shown as mean ± SEM, and statistical significance was calculated by 2-tailed, unpaired t test. *P<0.05, **P<0.01, ***P<0.001. BLCA, bladder cancer; ESCA, esophageal squamous cell carcinoma; HNSC, head and neck squamous cell carcinoma; KIRC, kidney renal clear cell carcinoma; KIRP, kidney renal papillary cell carcinoma; LUAD, lung adenocarcinoma; LUSC, lung squamous cell carcinoma; THCA, thyroid carcinoma; UCEC, uterine corpus endometrial carcinoma.

Supplementary Figure 6

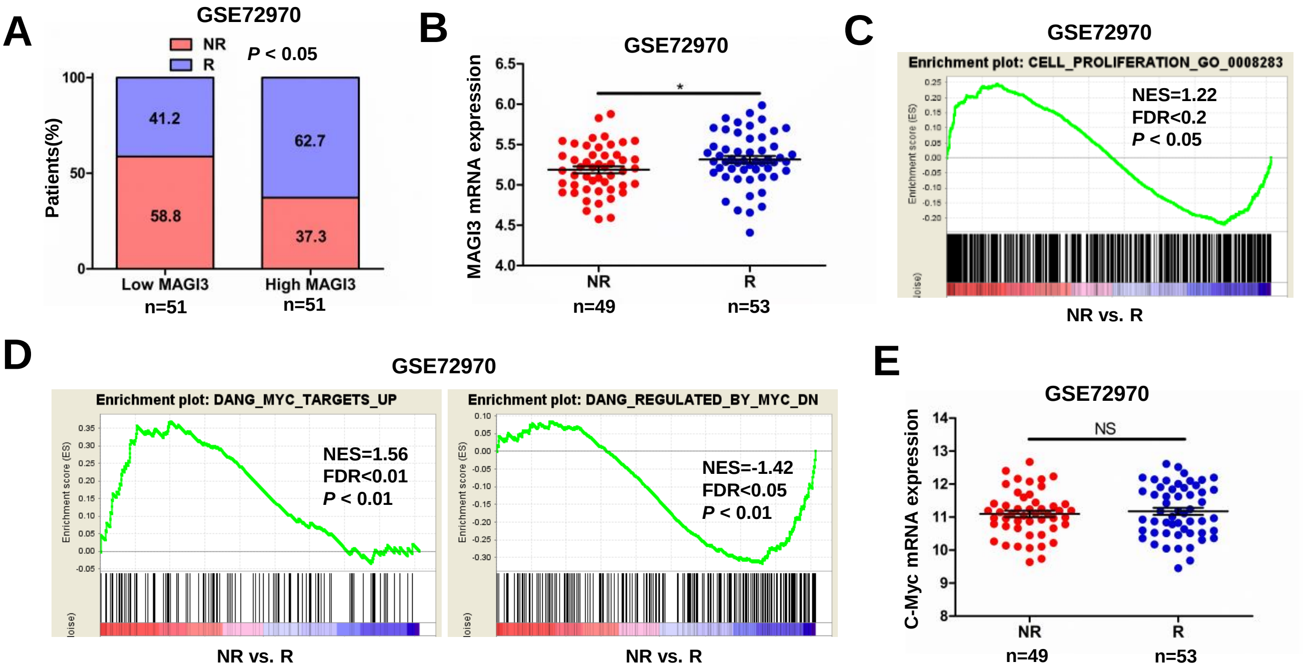

Figure S6. Loss of MAGI3 expression in CRC patients reduces sensitivity of chemotherapy by upregulation of c-Myc protein level and activation.
(A) The chi-square test showed that the CRC patients from dataset GSE72970 with high *MAGI3* mRNA level had high response ratio to chemotherapy. (B) Scatter plots of *MAGI3* mRNA expression in patients with non-response (NR) or response (R). (C and D) Enrichment plots of GSEA showed that the gene signatures of cell proliferation (C), c-Myc positive activation (D, left) were enriched in NR group and c-Myc negative regulated pathway (D, right) was enriched in R group. (E) Scatter plots of c-Myc mRNA expression in patients with non-response (NR) or response (R). Data were presented as the mean ± SEM, the chi-square test (A), 2-tailed, unpaired t test (B and E) were used to determine statistical significance. *P<0.05 and NS, no significance.
